# Evaluation of Buprenorphine Rotation in Patients Receiving Long-term Opioids for Chronic Pain: A Systematic Review

**DOI:** 10.1001/jamanetworkopen.2021.24152

**Published:** 2021-09-08

**Authors:** Victoria D. Powell, Jack M. Rosenberg, Avani Yaganti, Claire Garpestad, Pooja Lagisetty, Carol Shannon, Maria J. Silveira

**Affiliations:** 1 Palliative Care Program, Division of Geriatric and Palliative Medicine, University of Michigan, Ann Arbor; 2 Geriatrics Research, Education, and Clinical Center, LTC Charles S. Kettles Veterans Affairs (VA) Medical Center, Ann Arbor, Michigan; 3 Department of Physical Medicine and Rehabilitation, University of Michigan, Ann Arbor; 4 Department of Anesthesiology, University of Michigan, Ann Arbor; 5 Department of Physical Medicine and Rehabilitation, LTC Charles S. Kettles VA Medical Center, Ann Arbor, Michigan; 6 Department of Anesthesiology, LTC Charles S. Kettles VA Medical Center, Ann Arbor, Michigan; 7 Department of Internal Medicine, University of Michigan, Ann Arbor; 8 University of Michigan Medical School, Ann Arbor; 9 Center for Clinical Management and Research, Ann Arbor VA, Ann Arbor, Michigan; 10 Taubman Health Sciences Library, University of Michigan, Ann Arbor

## Abstract

**Importance:**

Individuals with chronic pain who use long-term opioid therapy (LTOT) are at risk of opioid use disorder and other harmful outcomes. Rotation to buprenorphine may be considered, but the outcomes of such rotation in this population have not been systematically reviewed.

**Objective:**

To synthesize the evidence on rotation to buprenorphine from full μ-opioid receptor agonists among individuals with chronic pain who were receiving LTOT, including the outcomes of precipitated opioid withdrawal, pain intensity, pain interference, treatment success, adverse events or adverse effects, mental health condition, and health care use.

**Evidence Review:**

PubMed, CINAHL, Embase, and PsycInfo were searched from inception through November 3, 2020, for peer-reviewed original English-language research that reported the prespecified outcomes of rotation from prescribed long-term opioids to buprenorphine among individuals with chronic pain. Two independent reviewers extracted data as well as assessed risk of bias and study quality according to the Preferred Reporting Items for Systematic Reviews and Meta-analyses (PRISMA) reporting guidelines. Quality of evidence was assessed with the Grading of Recommendations Assessment, Development and Evaluation (GRADE) system.

**Findings:**

A total of 22 studies were analyzed, of which 5 (22.7%) were randomized clinical trials, 7 (31.8%) were case-control or cohort studies, and 10 (45.5%) were uncontrolled pre-post studies, which involved 1616 unique participants (675 female [41.8%] and 941 male [58.2%] individuals). Six of the 22 studies (27.3%) were primary or secondary analyses of a large randomized clinical trial. Participants had diverse pain and opioid use histories. Rationale for buprenorphine rotation included inadequate analgesia, intolerable adverse effects, risky opioid regimens (eg, high dose and/or sedative coprescriptions), and aberrant opioid use. Most protocols were adapted from protocols for initiating treatment in patients with opioid use disorder and used buccal or sublingual buprenorphine. Very low-quality evidence suggested that buprenorphine rotation was associated with maintained or improved analgesia, with a low risk of precipitating opioid withdrawal. Steady-dose buprenorphine was better tolerated than tapered-dose buprenorphine. Adverse effects were manageable, and severe adverse events were rare. Only 2 studies evaluated mental health outcomes, but none evaluated health care use. Limitations included a high risk of bias in most studies.

**Conclusions and Relevance:**

In this systematic review, buprenorphine was associated with reduced chronic pain intensity without precipitating opioid withdrawal in individuals with chronic pain who were receiving LTOT. Future studies are necessary to ascertain the ideal starting dose, formulation, and administration frequency of buprenorphine as well as the best approach to buprenorphine rotation.

## Key Points

**Question:**

Is rotation to buprenorphine from full μ-opioid receptor agonists associated with improved pain-related outcomes and acceptable adverse effects in patients with chronic pain and long-term use of opioids?

**Findings:**

In this systematic review of 22 studies that addressed prespecified outcomes of rotation to buprenorphine, low-quality evidence suggested that buprenorphine rotation was associated with reduced pain without precipitating opioid withdrawal or other serious adverse effects.

**Meaning:**

These findings suggest that buprenorphine rotation may be a viable option for mitigating the harms of long-term opioid therapy in individuals with chronic pain who were receiving unsafe opioid analgesic regimens; further studies are needed to examine the best way to accomplish buprenorphine rotation.

## Teaser

This systematic review reports the potential for withdrawal, pain level, treatment success, adverse events, and other outcomes of discontinuing long-term opioid therapy and switching to buprenorphine.

## Introduction

Some individuals with chronic pain who use long-term opioid therapy (LTOT) have reported modest improvements in pain and functioning^1,2^; however, LTOT confers risks, including opioid misuse, suppression of immune and endocrine function, and accidental overdose.^2,3,4,5^ When risks outweigh benefits, tapering or discontinuing LTOT is recommended.^6^ In practice, however, tapering is challenging^7,8^ and may result in increased pain and psychological distress.^9,10^ One possible alternative to tapering LTOT is rotation to buprenorphine.^8,11^

As a partial μ-opioid receptor (MOR) agonist, buprenorphine is associated with less respiratory depression, fatal overdose, and overall mortality^12,13,14,15,16,17^ as well as fewer adverse effects than full MOR agonists (ie, morphine sulfate or oxycodone hydrochloride).^18,19,20,21,22^ Drug clearance does not change with age, renal impairment, or mild hepatic impairment.^23,24,25^ These properties make buprenorphine an attractive replacement for other opioids. However, rotation to buprenorphine is not without risk.

Buprenorphine has the potential to precipitate severe opioid withdrawal because of its high MOR affinity but partial agonist activity.^26^ Older data that showed a ceiling on certain subjective outcomes of buprenorphine^13^ have raised concern that rotation to buprenorphine could theoretically worsen pain for individuals who are benefiting from full MOR agonists for analgesia, even though more recent data suggest otherwise.^27^ The risks of buprenorphine have been well established in patients with opioid use disorder (OUD) without chronic pain, and the risks primarily include precipitated opioid withdrawal and potential for misuse and diversion.^26^ It is hypothesized that these risks would be similar in patients with chronic pain who were prescribed LTOT. However, this hypothesis has not been well characterized.

Moreover, little guidance is currently available on how to conduct such a rotation in this population; protocols developed for individuals with OUD may be inappropriate. Therefore, we conducted a systematic review of the literature to synthesize the evidence on rotation to buprenorphine from full MOR agonists among individuals with chronic pain receiving LTOT, including the outcomes of precipitated opioid withdrawal, pain intensity, pain interference, treatment success, adverse events (AEs) or adverse effects, mental health condition, and health care use.

## Methods

We followed the Preferred Reporting Items for Systematic Reviews and Meta-analyses (PRISMA) reporting guideline. One of us (C.S.) searched MEDLINE (PubMed), CINAHL, Embase, and PsycInfo from inception to November 3, 2020, for peer-reviewed, original English-language research that addressed the prespecified outcomes of rotation to buprenorphine from prescribed long-term opioids among individuals with chronic pain. We used the following concepts: chronic pain, opioids, buprenorphine, and pain management (eAppendix in the Supplement). Articles were exported to DistillerSR software (Evidence Partners Incorporated). The systematic review protocol was registered in PROSPERO (CRD42020173272). This study was exempt from institutional review board in accordance with §46.104 (d)(3)(i)(A) of the Basic US Department of Health and Human Services Policy for Protection of Human Research Subjects,^28^ because it used deidentified, publicly available data.

### Study Eligibility and Selection

Every level of the articles required the input of 2 reviewers (including V.D.P., J.M.R., M.J.S., A.Y., and C.G.), who made the decision on study progression. Inclusion criteria were studies (1) that enrolled participants who had chronic pain, were prescribed full MOR opioids (eg, morphine and oxycodone) for pain on most of the past 90 days, and were initiated on buprenorphine and (2) that reported prespecified outcomes of interest (eFigure in the Supplement). It is challenging to distinguish LTOT-induced physical dependence or tolerance to opioids from OUD that developed from prescription opioid use.^29,30^ Discrimination is further complicated by the removal of dependence and tolerance from the OUD diagnostic criteria in the *Diagnostic and Statistical Manual of Mental Disorders*, Fifth Edition (*DSM-5*).^31^ For these reasons, the association between LTOT for chronic pain and OUD is complex. Thus, we included studies of individuals with chronic pain who were prescribed any full MOR agonist for the long term, regardless of the presence of OUD diagnosis. We excluded studies whose participants primarily consumed nonprescribed opioids.

### Data Extraction, Quality Assessment, and Synthesis

Data extracted included study design, pain characteristics, length of time using opioids, and baseline opioid dose. Outcomes of rotation to buprenorphine included precipitated opioid withdrawal, pain intensity or severity, pain interference, treatment success (eg, completion of protocol, willingness to continue buprenorphine long term, and reduced interest in additional opioids), mental health condition (eg, depression and insomnia), AEs or adverse effects, and health care use (eg, hospitalizations and outpatient visits). We focused on withdrawal symptoms precipitated by initiation of buprenorphine rather than by discontinuation of the original opioid (if that distinction could be made). We also extracted details regarding buprenorphine conversion protocols, including length of time between the most recent baseline opioid dose and the initial buprenorphine administration, dose, formulation, and frequency (eg, twice daily).

Risk of bias and study quality were assessed using the Cochrane Collaboration tool^32^ for randomized clinical trials (RCTs) and the Newcastle-Ottawa Scale^33^ for cohort and case-control studies. All uncontrolled observational studies (ie, pre-post studies) were considered to have inherently high risk of bias. Data were synthesized using the Grading of Recommendations Assessment, Development and Evaluation (GRADE) criteria^34^ for each outcome. Heterogeneity precluded quantitative meta-analysis.

## Results

A total of 2196 unique publications were identified through literature search, of which 128 full-text articles met the initial inclusion criteria. Of the 128 articles, 22 qualified for inclusion in this study (Figure). These articles represented 5 RCTs (22.7%),^27,35,36,37,38^ 7 case-control or cohort studies (31.8%),^39,40,41,42,43,44,45^ and 10 uncontrolled pre-post studies (45.5%),^46,47,48,49,50,51,52,53,54,55^ which involved a total of 1642 unique participants (Table 1). However, Roux et al^36^ randomized 51 participants but included only 25 in their analysis, decreasing the total number of participants to 1616 (675 female [41.8%] and 941 male [58.2%] individuals). Six of the 22 key studies (27.3%)^38,41,42,43,44,45^ were primary or secondary analyses of the Prescription Opioid Addiction Treatment Study (POATS).^56^

**Figure.**
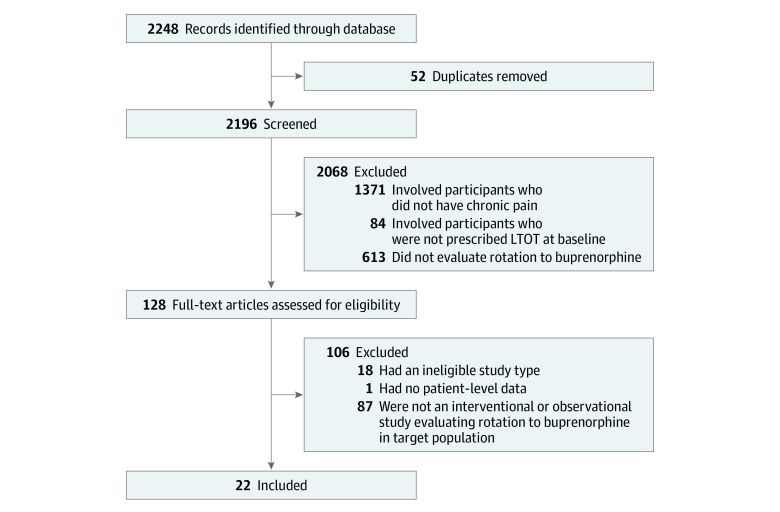
PRISMA Study Flow Diagram LTOT indicates long-term opioid therapy.

**Table 1.** Studies of Buprenorphine Rotation in Patients with Chronic Pain on Long-term Opioid Therapy

Source	Setting and select inclusion criteria	Design	Buprenorphine formulation	Sample size, including control participants (if any)	Outcomes						
					Pain intensity or severity	Pain interference	Precipitated opioid withdrawal	Treatment success	Adverse effects or adverse events	Mental health condition	ROB or quality assessment (instrument used)
Aurilio et al,^46^ 2009	Outpatient; participants had chronic cancer pain with inadequate analgesia and intolerable opioid adverse effects; no SUD	Uncontrolled pre-post study	Transdermal patch	32	Yes	NA	NA	NA	Yes	NA	Inherently high ROB attributed to study design
Baron et al,^39^ 2006	Inpatient setting for detoxification and/or buprenorphine initiation and then outpatient follow-up; participants had inadequate analgesia from current opioid regimen; no concern for overuse, abuse, or addiction	Cohort study	Sublingual without naloxone hydrochloride dihydrate	23	Yes	NA	NA	NA	NA	NA	6 of 9 (NOS)^a^
Berland et al,^47^ 2013	Two-center inpatient or outpatient setting for buprenorphine initiation and then outpatient follow-up; participants had worsening pain and function despite increasing long-term opioid doses; 18 participants (24%) had “concern for addiction”	Uncontrolled pre-post study	Combination of formulations	76	Yes	Yes	Yes	Yes	Yes	NA	Inherently high ROB attributed to study design
Blondell et al,^35^ 2010	Inpatient setting for buprenorphine initiation and stabilization and then outpatient follow-up; participants had chronic, nonmalignant pain and met *DSM-IV* criteria of opioid dependence to prescribed opioids	RCT	Sublingual tab or film with naloxone	12	Yes	Yes	NA	Yes	NA	NA	High (Cochrane Collaboration tool)^b^
Daitch et al,^49^ 2012	Single-center pain clinic; all participants had inadequately controlled or worsening chronic pain and receiving LTOT	Uncontrolled pre-post study	Sublingual tab or film with naloxone	104	Yes	NA	NA	NA	Yes	NA	Inherently high ROB attributed to study design
Daitch et al,^50^ 2014	Single-center pain clinic; all participants had high-dose opioids prescription (≥200 MME) for chronic pain	Uncontrolled pre-post study	Sublingual without naloxone	35	Yes	NA	Yes	NA	NA	NA	Inherently high ROB attributed to study design
Freye et al,^51^ 2007	Mixed settings; all participants were prescribed >120 mg morphine sulfate/d with inadequate analgesia and/or severe adverse effects	Uncontrolled pre-post study	Transdermal patch	42	Yes	NA	NA	NA	Yes	Yes	Inherently high ROB attributed to study design
Malinoff et al,^52^ 2005	Single-center pain clinic; all participants had worsening chronic pain despite escalating opioid doses; 8.4% of participants met *DSM-IV* criteria for opioid dependence diagnosis	Uncontrolled pre-post study	Sublingual tab or film with naloxone	95	Yes	NA	NA	Yes	Yes	NA	Inherently high ROB attributed to study design
Neumann et al,^37^ 2020	Primary care–like outpatient; all participants had postsurgical chronic back pain and met *DSM-IV* criteria for opioid dependence to prescribed opioids	RCT	Sublingual tab or film with naloxone	19	Yes	Yes	NA	Yes	Yes	Yes	High (Cochrane Collaboration tool)^b^
Pade et al,^48^ 2012	Specialty single-center clinic; participants were veterans who were referred to the clinic for combined chronic pain, high-risk opioid use (ie, high dose or combined with sedating medications), and/or co-occurring SUD	Uncontrolled pre-post study	Sublingual tab or film with naloxone	143	Yes	NA	NA	Yes	NA	NA	Inherently high ROB attributed to study design
Rosenblum et al,^54^ 2012	Outpatient single-center pain clinic; participants with chronic pain were prescribed LTOT; all participants had aberrant opioid-related behavior but did not meet current *DSM-IV* criteria for any SUD diagnosis	Uncontrolled pre-post study	Sublingual tab or film with naloxone	12	Yes	Yes	Yes	Yes	Yes	NA	Inherently high ROB attributed to study design
Roux et al,^36^ 2013	Inpatient research unit; all participants had chronic, nonmalignant pain and met *DSM-IV* criteria for opioid dependence diagnosis but were not seeking treatment	RCT	Sublingual tab or film with naloxone	51	Yes	NA	Yes	Yes	Yes	NA	High (Cochrane Collaboration tool)^b^
Streltzer et al,^55^ 2015	Outpatient single-center psychiatrist-run pain clinic; participants were using LTOT and referred by primary care for difficult-to-control chronic pain; all participants met *DSM-IV* criteria for opioid dependence diagnosis but frequently took opioids as prescribed	Uncontrolled pre-post study	Sublingual tab or film with naloxone	43	NA	NA	NA	Yes	NA	NA	Inherently high ROB attributed to study design
Sturgeon et al,^40^ 2020	Outpatient single-center specialty opioid refill clinic for individuals with chronic pain who were prescribed a high dose of LTOT; all participants were initially offered tapering and were rotated to buprenorphine if they were not able to tolerate tapering to ≤90 MME or demonstrated aberrant opioid-related behavior	Cohort study	Sublingual without naloxone	240	Yes	NA	NA	Yes	NA	NA	6 of 9 (NOS)^a^
Tang et al,^53^ 2020	Single-center inpatient setting in which individuals were initiated on buprenorphine while hospitalized and followed up as outpatients; all participants had either OUD or chronic pain–related opioid dependence	Uncontrolled pre-post study	Combination of formulations	23	NA	NA	Yes	Yes	NA	NA	Inherently high ROB attributed to study design
Webster et al,^27^ 2016	Inpatient research unit; participants had chronic pain and physical opioid dependence (developed withdrawal symptoms with naloxone challenge) but no active SUD	RCT	Buccal	39	Yes	NA	Yes	Yes	Yes	NA	High (Cochrane Collaboration tool)^b^
**POATS articles** ^**c**^											
Griffin et al,^44^ 2016	Secondary analysis of POATS; limited to participants with chronic pain who participated in extended, 12-wk buprenorphine treatment	Case-control study	Sublingual tab or film with naloxone	148	Yes	NA	NA	NA	NA	NA	7 of 9 (NOS)^a^
Nielsen et al,^41^ 2014	Secondary analysis of POATS; limited to participants who used methadone, extended-release oxycodone, immediate-release oxycodone, or hydrocodone before buprenorphine rotation and who had both predosing and postdosing withdrawal scores available	Case-control study	Sublingual tab or film with naloxone	569	NA	NA	Yes	NA	NA	NA	9 of 9 (NOS)^a^
Weiss et al,^45^ 2014	Secondary analysis of POATS; limited to participants who were randomized in the extended, 12-wk buprenorphine treatment phase; 38.3% reported current chronic pain	Case-control study	Sublingual tab or film with naloxone	360	NA	NA	NA	Yes	NA	NA	8 of 9 (NOS)^a^
Weiss et al,^38^ 2011	Primary analysis of the multisite RCT; outpatient setting; all participants with self-identified dependence on prescription opioids (n = 274 [42%]) had current chronic pain	RCT	Sublingual tab or film with naloxone	653	NA	NA	NA	Yes	Yes	NA	Some concerns (Cochrane Collaboration tool)^b^
Worley et al,^43^ 2017	Secondary analysis of POATS; limited to participants with chronic pain who participated in the extended, 12-wk buprenorphine treatment phase and who completed at least 1 outcome assessment during the taper phase	Case-control study	Sublingual tab or film with naloxone	125	Yes	NA	NA	NA	NA	NA	9 of 9 (NOS)^a^
Worley et al,^42^ 2015	Secondary analysis of POATS; limited to participants with chronic pain who participated in the extended, 12-wk buprenorphine treatment phase	Case-control study	Sublingual tab or film with naloxone	149	Yes	NA	NA	Yes	NA	NA	9 of 9 (NOS)^a^

Abbreviations: *DSM-IV, Diagnostic and Statistical Manual of Mental Disorders* (Fourth Edition); LTOT, long-term opioid therapy; MME, oral morphine milligram equivalent; NA, not applicable; NOS, Newcastle-Ottawa Scale; OUD, opioid use disorder; POATS, Prescription Opioid Addiction Treatment Study; RCT, randomized clinical trial; ROB, risk of bias; SUD, substance use disorder.

^a^ NOS case-control and cohort studies score range: 0-9, with higher scores indicating less ROB.

^b^ Cochrane Collaboration tool assesses the ROB (range: low, unclear, or high) in 6 domains (selection, performance, detection, attrition, reporting, and other). Overall ROB was scored from some concerns to high, with more domains that are scored high risk indicating higher overall ROB.

^c^ Primary analysis was based on Weiss et al^38^; other studies were secondary analyses that met the inclusion criteria.

Full study details are reported in eTable 1 in the Supplement, details of GRADE scoring are reported in eTable 2 in the Supplement, and details of the risk-of-bias assessment are shown in eTables 3 to 5 in the Supplement. The summary of findings and risk-of-bias assessment is presented in Table 2.

**Table 2.** Summary of Findings and Grading of Recommendations Assessment, Development and Evaluation (GRADE)^a^

Outcome	No. of studies	Study design (No. of studies)	GRADE score^b^	Results
Precipitated opioid withdrawal	7	RCT (n = 2)^27,36^	Low	Precipitated opioid withdrawal was rare (incidence of 3%-6%) and was generally mild when present In a few cases, withdrawal was severe; risk appeared to be higher when participants used high opioid doses before rotation Most studies required the presence of mild opioid withdrawal before induction with buprenorphine Heterogeneity was observed in assessment of withdrawal and study populations
		Controlled observational (n = 1)^41^	Low	
		Uncontrolled pre-post (n = 4)^47,50,53,54^	Very low	
Pain intensity or severity	17	RCT (n = 4)^27,35,36,37^	Low	Improved analgesia was observed after rotation in 12 of 17 studies Effect size was attenuated in studies with control groups Evidence of dose-response was found (higher buprenorphine doses were associated with better pain control) Analgesic effect may be attenuated in those who used high doses before switching Heterogeneity of measurement (timing and instruments), study populations, and rationale for rotation were observed
		Controlled observational (n = 5)^39,40,42,43,44^	Very low	
		Uncontrolled pre-post (n = 8)^46,47,48,49,50,51,52,54^	Very low	
Pain interference	4	RCT (n = 2)^35,37^	Very low	Improvement in function observed in some individuals after rotation Heterogeneity was observed in study populations and instruments measuring function
		Uncontrolled pre-post (n = 2)^47,54^	Very low	
Treatment success: completion of protocol or continuation of treatment	14	RCT (n = 5)^27,35,36,37,38^	Very low	Protocol completion rates and willingness to continue in treatment were higher when buprenorphine was not tapered off Retention rates ranged from 33% to 93% Follow-up periods were often nonsystematic outside of RCTs
		Controlled observational (n = 3)^40,42,45^	Very low	
		Uncontrolled pre-post (n = 6)^47,48,52,53,54,55^	Very low	
Adverse effects or adverse events	10	RCT (n = 4)^27,36,37,38^	Very low	Adverse effects were common, usually mild, and similar to other opioids Occasionally, adverse effects were severe and required drug discontinuation No deaths or overdoses were attributed to buprenorphine in any study
		Uncontrolled pre-post (n = 6)^46,47,49,51,52,54^	Very low	
Mental health condition	2	RCT (n = 1)^37^	Very low	One RCT found improvement in depressive symptoms at 6 months; 1 uncontrolled study found improvement in sleep quality
		Uncontrolled pre-post (n = 1)^51^	Very low	
Health care use	0	NA	NA	No studies examined this outcome

Abbreviations: NA, not applicable; RCT, randomized clinical trial.

^a^ Controlled observational studies included cohort and case-control study designs.

^b^ GRADE score range: very low to high, with higher scores indicating higher-quality body of evidence.

### Study Populations

Identified studies included participants with mostly chronic musculoskeletal pain but also neuropathic pain and fibromyalgia. In general, participants had diverse pain and opioid use histories. One study exclusively focused on those with chronic cancer pain,^46^ whereas several studies excluded those with cancer.^35,36,47,48^ Participants were rotated to buprenorphine for various indications, including inadequate analgesia with escalating opioid doses, intolerable adverse effects, and risky regimens (eg, high dose and/or coprescription with benzodiazepines).^39,40,46,47,49,50,51,52,53^ Other reasons included aberrant opioid use.^35,36,37,38,48,54,55^

Opioid use disorder was present in some participants in 13 of 22 studies (59.1%),^35,36,37,38,41,42,43,44,45,47,48,52,53^ whereas in 4 of 22 studies (18.2%)^27,39,40,46,49,50,51,54,55^ OUD was an exclusion criterion; however, in 2 of these studies, participants either had some aberrant opioid-related behavior^54^ or were opioid dependent by naloxone challenge.^27^ Although OUD presence was unspecified in 5 of 22 studies (22.7%), problematic behavior or opioid dependence was noted, even when participants used opioids exactly as prescribed.^55^ The baseline daily opioid doses were highly variable (total range, 10-3200 oral morphine milligram equivalents [MMEs]; mean daily dose range, 60-500 MME). A daily dose greater than 1000 MME was prescribed to some individuals in several studies.^39,40,50,55^

### Rotation Protocols

Buprenorphine rotation protocols were diverse. Of the 17 unique protocols that were identified, 9 were adaptations of protocols that were originally intended for buprenorphine induction in individuals with OUD without chronic pain. These 9 protocols required the presence of mild opioid withdrawal symptoms before the first buprenorphine dose and/or low starting doses, reassessment of withdrawal symptoms, and additional dose administration if needed.^35,37,38,40,48,49,50,52,54^ Eight protocols instructed individuals to stop opioids 8 to 24 hours before the first buprenorphine dose,^27,38,40,48,49,50,52,53^ and 3 protocols required an overnight waiting period.^35,37,54^ Individuals who were switching from methadone hydrochloride or transdermal fentanyl were instructed to wait longer (about 36-72 hours).^38,47,48,49,50,54^

In 2 protocols, participants were randomized to structured buprenorphine tapering conditions^35,56^; in 1 study, participants were allowed to switch to the steady-dose group if they could not tolerate the tapering.^35^ Another protocol required that patients were unable to taper their previous opioid use before being offered buprenorphine rotation.^40^ In 10 of 14 protocols without a structured tapering condition, rotation to buprenorphine was reported as completely voluntary.^27,36,39,46,47,49,50,52,54,55^ For the 4 remaining protocols, it was not specified whether rotation was voluntary or a contingency of ongoing treatment.^37,48,51,53^

Buccal or sublingual buprenorphine was used exclusively in 13 protocols, often in combination with naloxone hydrochloride dihydrate.^27,35,36,37,38,39,40,48,49,50,52,54,55^ Transdermal buprenorphine was used exclusively in 2 protocols.^46,51^ Two protocols provided multiple buprenorphine formulations.^47,53^ Buprenorphine sublingual or buccal dosing varied widely, from a first-day total dose of 600 μg^27^ to a maximum possible dose of 32 mg.^49,50^ Nine protocols divided administration into 2 to 4 doses per day.^27,35,36,37,39,47,48,52,54^ Transdermal buprenorphine dosing also varied widely, from 5 μg/h^53^ to 140 μg/h.^51^ The study by Tang et al^53^ applied a microdose approach to minimize anxiety and withdrawal symptoms, whereby a lower-dose transdermal formulation of buprenorphine served as a bridge to higher sublingual doses. Ten protocols allowed additional medication to ease symptoms of withdrawal, although specific medications varied.^27,35,36,37,39,40,46,47,50,54^ eTable 6 in the Supplement describes the buprenorphine rotation protocols.

### Precipitated Opioid Withdrawal

Precipitated opioid withdrawal (defined as worsening withdrawal symptoms after receipt of the first dose of buprenorphine) was rare, according to very low– to low-quality evidence. The 7 studies that examined this outcome consisted of 2 small RCTs,^27,36^ 1 with a case-control design (secondary analysis of a large RCT),^41^ and 4 uncontrolled pre-post studies.^47,50,53,54^ Withdrawal symptoms were measured in 6 of 7 studies using the Clinical Opiate Withdrawal Scale (score ranges: 13-24, indicating moderate withdrawal; 25-36, indicating moderately severe withdrawal; and >36, indicating severe withdrawal^57^) or the Subjective Opiate Withdrawal Scale (score ranges: 1-10, indicating mild withdrawal; 11-20, indicating moderate withdrawal; and 21-30 indicating severe withdrawal^58^). Symptoms were typically measured within an hour before or after induction; 1 study took multiple measurements for the 12 hours after buprenorphine was initiated.^27^

The incidence of precipitated withdrawal ranged from 3% to 6%.^27,41^ Assessed with the Clinical Opiate Withdrawal Scale, the mean severity was in the low to moderate range (mean [SD], 13.6 [2.9]).^41^ The risk of withdrawal symptoms was greater when baseline opioid dose was higher.^50,54^ The specific full MOR agonist or its formulation (extended release vs immediate release) was not associated with withdrawal symptoms.^41^

### Pain Severity and Interference

Rotation to buprenorphine was associated with decreased pain severity in 12 of 17 studies (70.6%).^35,36,42,43,46,47,48,49,50,51,52,54^ For example, Roux et al^36^ found that higher doses of buprenorphine were associated with better pain control among participants. Under the highest dose condition (16 mg vs 2 mg), the McGill Pain Questionnaire scores significantly decreased (odds ratio, 0.42; 95% CI, 0.20-0.90).^36^ Under any dose, the median McGill Pain Questionnaire score decreased from 38 to 21 (*P* < .001).^36^ In the remaining 5 of 17 studies, no association was found between buprenorphine and pain.^27,37,39,40,44^ It was suggested that individuals who were rotating from higher doses (>200 MME) of full MOR agonists experienced less analgesia after rotation to buprenorphine; however, the evidence was very low quality.^49,54^

The evidence supporting an association between buprenorphine and pain intensity was very low to low quality. The 17 studies that examined this outcome included 4 small RCTs,^27,35,36,37^ 5 controlled observational studies,^39,40,42,43,44^ and 8 uncontrolled observational studies.^46,47,48,49,50,51,52,54^ Substantial heterogeneity in timing of pain assessment was found, with some studies assessing pain immediately after buprenorphine was administered^27,36^ and other studies assessing pain only at the end of the study period^37,49,50^ or after buprenorphine was discontinued.^39^ Only 7 studies used detailed, validated instruments, such as the Brief Pain Inventory^37,42,43,44,54^ and McGill Pain Questionnaire.^36,46^ Other studies used unidimensional pain assessments, such as the visual analog scale or numeric rating scale,^27,39,40,48,49,50,52^ or unvalidated measures.^35,47,51^

Pain interference may decrease after rotation to buprenorphine, but only 4 studies examined this outcome and all had high risk of bias.^35,37,47,54^ Neumann et al^37^ found a 21.4% improvement in self-reported function overall but was underpowered to detect a difference that was specific to the buprenorphine group. Studies used varied measures of pain interference. One small RCT used the Roland-Morris Disability Questionnaire,^37^ 1 observational study used the Brief Pain Inventory functional subscale,^54^ and 2 studies used nonvalidated measures.^35,47^ Follow-up periods and assessment timing also varied. Overall, the evidence quality was very low.

### Treatment Success 

The evidence suggested that rotation to buprenorphine can be accomplished successfully in participants with chronic pain who were receiving LTOT.^27,35,36,37,38,40,47,48,52,53,55^ Success rates were higher when buprenorphine was used long term (53%-83%)^35,37^ rather than tapered off (0%-49%).^35,38^ Blondell et al^35^ found that no participants in the tapering group of a small RCT were able to complete the taper, whereas most participants in the steady-dose group completed the prespecified follow-up period. Similarly, the POATS^38^ and subsequent secondary analysis^42^ found that participants with chronic pain were less likely to use additional opioids during the steady-dose phase than after being tapered off of buprenorphine, especially if their pain was controlled with buprenorphine.

Most studies that examined the success of buprenorphine rotation were of very low quality. The RCTs found high rates of protocol completion,^27,36,38^ willingness to continue using buprenorphine for the long term,^35,37^ and reduced need for additional opioids while receiving steady doses of buprenorphine.^36,38^ These studies were all small, with the exception of the POATS.^38^ Studies had variable and often nonsystematic follow-up periods, which precluded the rigorous assessment of protocol completion or continuation of treatment. Substantial indirectness was found across the body of evidence, with an assortment of primary outcomes, study populations, and length of time that buprenorphine was administered.

### Adverse Events or Adverse Effects

Rotation to buprenorphine was associated with little harm to patients, as suggested by the 4 RCTs^27,36,37,38^ and 6 uncontrolled observational studies^46,47,49,51,52,54^ that reported AEs and/or adverse effects. The most common adverse effects included headache, gastrointestinal symptoms (ie, nausea or vomiting, constipation, and appetite changes), lightheadedness or dizziness, sedation, and sleep disruption.^36,38,49,52,54^ Skin irritation was common in participants who were using transdermal buprenorphine.^51^

The severity of adverse effects was, overall, mild and associated with the opioid class. Rarely were AEs or adverse effects severe enough to require the discontinuation of buprenorphine; for example, the POATS primary analysis reported a 2.5% discontinuation rate.^38^ When AEs or adverse effects were examined over time, they generally decreased in intensity and, in some cases, resolved completely within a week.^46,54^ Yet, some adverse effects persisted for at least 6 months.^37^ In 1 study, the US Food and Drug Administration placed further recruitment on hold because of severe AEs, such as hospitalization for intense withdrawal symptoms and pain, in 7 of 12 participants.^54^ This small pilot study was focused on developing a buprenorphine transition protocol that underwent multiple iterations during the study course. Its investigators noted that the protocol, which did not take into consideration participants’ baseline opioid formulation or dose, may not have allowed adequate flexibility for those who were using the highest and lowest MME doses.^54^ Heavy sedation was reported by only 1 study in a single patient.^36^ No accidental or intentional overdoses or deaths were attributed to buprenorphine in any studies; a single overdose-related death was reported in 1 participant that occurred months after discontinuing buprenorphine.^55^

### Mental Health Condition and Health Care Use

There was insufficient evidence to definitively support an association between outcomes of mental health conditions (eg, depression, anxiety, and insomnia) and buprenorphine rotation. Only 2 studies reported mental health outcomes^37,51^ and both had high risk of bias. Nevertheless, 2 studies suggested that buprenorphine rotation was associated with improved mental health outcomes. Neumann et al^37^ reported a significant decrease in Beck Depression Inventory scores (from 21.2 to 16.2; *P* = .01), which was consistent with a change from moderate to mild depression at 6-month follow-up. Freye et al^51^ reported that, after rotation to buprenorphine from high-dose morphine, 74% of participants experienced improved quality of sleep vs 14% of participants before the rotation.

No studies assessed the implication of buprenorphine rotation for health care use, such as outpatient clinic visits, emergency department visits, or hospitalizations.

## Discussion

We systematically reviewed the literature regarding rotation to buprenorphine in individuals with chronic pain who used LTOT. We found 22 studies that evaluated at least 1 prespecified outcome. Although most studies had a high risk of bias, limiting the strength of the present study’s conclusions, several important findings emerged.

Buprenorphine appeared to be not inferior to full MOR agonists in controlling pain. Continuing buprenorphine rather than tapering it off was associated with higher rates of protocol completion and lower use of additional opioids. Although many approaches to buprenorphine rotation are available, most studies relied on protocols that were adapted from those that are used to induct patients with OUD to buprenorphine, which require that opioid withdrawal symptoms be present before the administration of the first dose of buprenorphine. Previous studies also supported the safety of buprenorphine. Severe AEs were rare, and the adverse effects, although more common, were manageable. The incidence of precipitated opioid withdrawal was low (3%-6%).

We believe that this systematic review adds to the growing body of literature that suggests that buprenorphine is safe to use in multiple populations, including those with opioid dependence and chronic pain.^16,59,60^ In contrast, long-term full MOR agonists present significant risks for harmful or fatal overdose, all-cause mortality, and morbidity.^1,2,6^ Thus, this synthesis supports the hypothesis that rotation from full MOR agonists to buprenorphine would reduce harm, although more research is needed into the best way to complete such rotation.

Rotation to buprenorphine was not associated with changes to patients’ pain control. In most studies, buprenorphine was associated with reduced pain severity, although the mechanism for this finding is not clear. It is possible that opioid rotation in general (through incomplete cross-tolerance to a different opioid), rather than rotation to buprenorphine specifically, was a factor.^61,62,63^ Alternatively, reduced pain might be associated with buprenorphine’s unique role in opioid-induced hyperalgesia.^17,64^ Previous studies have reported the antihyperalgesic outcomes of buprenorphine compared with other full MOR agonists in animal models and in humans.^17,65,66,67,68^

Participants were much more likely to complete study protocols, remain in treatment, avoid additional opioid use, and achieve analgesia if they continued to receive stable doses of buprenorphine after rotation rather than tapering off of buprenorphine. This outcome was especially apparent in individuals with co-occurring OUD and chronic pain,^35,38,43^ which is consistent with the literature on OUD without chronic pain that found a greatly increased risk of return to illicit opioid use after buprenorphine taper.^69,70,71^ Findings from the present study suggest that continuing buprenorphine can be a successful strategy for patients with chronic pain who are unable or unwilling to taper opioids entirely. However, future studies should compare buprenorphine maintenance with tapering directly and should follow up for longer periods.

These findings highlighted not only the many benefits of buprenorphine rotation but also that the state of the science is in its early stages. We did not identify an optimal approach for rotation from full MOR agonists. The most common method of rotation involved a protocol adapted from OUD that used sublingual buprenorphine.^26^ This method required cessation of opioids for at least 8 hours and up to 72 hours until mild opioid withdrawal symptoms developed, followed by administration of an initial, low dose of buprenorphine and assessment for precipitated opioid withdrawal. Few harms have been reported with this approach, but it needs to be examined before widespread adoption for several reasons. First, inducing even mild withdrawal symptoms before initiating buprenorphine is likely to exacerbate pain and produce emotional distress.^72,73^ Second, the time, resources, and expertise needed to support and monitor an individual who is going through withdrawal while initiating buprenorphine may exceed the time, resources, and expertise that many outpatient primary care or pain clinics have at their disposal. Thus, a mechanism for switching to buprenorphine with little to no opioid withdrawal symptoms is preferable, and its development should be prioritized in future research. Microdosing (or microinduction) is an alternative approach that involves the overlapping use of low-dose sublingual or transdermal buprenorphine as a bridge to higher doses while full MOR agonists are cross-tapered.^53,74,75,76,77,78^ Given that this is a relatively new method, more research is needed to assess its superiority over other approaches.

In addition to the various protocols for rotation, there was a wide range of baseline full MOR agonist doses, starting buprenorphine doses, and buprenorphine formulations across studies. A possible explanation for this diversity is the lack of reliable equianalgesic conversions from full MOR agonists to buprenorphine; transdermal buprenorphine equipotency ratios with oral morphine have been reported from 70:1 to 115:1.^79,80,81,82^ There is even less consistency regarding the equipotency ratios in sublingual and buccal buprenorphine given that bioavailability varies from 30% to 60% depending on the specific product and individual variation.^64,83^ Future research should address the optimal starting dose, formulation, and administration frequency of buprenorphine that are based on baseline total daily dose, ideally in the form of a standard equipotency ratio. The flexibility in regimen and the ability to frequently adjust doses and timing will be important considerations.

Moreover, the populations studied were highly diverse. Participants demonstrated a wide spectrum of problematic opioid use, reflecting the reality of clinical practice.^84,85^ Although several studies excluded individuals with evidence of any aberrant use, most studies included at least some participants with opioid misuse, opioid dependence, or OUD. Diagnosing OUD in the context of prescription-only opioid use for chronic pain is challenging because no clear consensus exists on how to define and recognize OUD in this setting.^86^ Problematic behaviors (eg, preoccupation with receiving opioids and repeated requests for early refills or dose increases) in this population are common but may not be sufficient to meet the *DSM-5* criteria for a diagnosis of OUD.^86,87^ This hypothesis was reflected in several studies included in the present systematic review, which focused on participants with aberrant use but excluded those with OUD.^54,55^

We believe that the diversity of these participants increases confidence that buprenorphine rotation may be a viable strategy for mitigating the harms of LTOT across a spectrum of individuals with chronic pain who receive it. This diversity also highlights the need for future research into whether buprenorphine might be helpful in certain populations with chronic pain who use LTOT and do not meet the *DSM-5* criteria for an OUD diagnosis but show aberrant use or misuse.

### Limitations

This study has several limitations. First, most studies in this systematic review were of low quality, primarily because of their design. Of the 22 included studies, only 5 were RCTs^27,35,36,37,38^; 7 had a case-control or cohort design^39,40,41,42,43,44,45^; and 10 were uncontrolled pre-post assessments of a single clinic’s experience.^46,47,48,49,50,51,52,53,54,55^ These studies had various follow-up periods that were not always prespecified or systematic. Second, there was heterogeneity in outcomes and measures. Many studies used brief, unidimensional measures of pain and some used unvalidated measures. Third, not all outcomes of importance were assessed. For example, pain-related interference, which is considered to be an outcome that is on par with pain intensity,^88^ was rarely measured. Similarly, depression and sleep disturbance, which are associated with chronic pain,^89,90,91^ were assessed in only 1 study each. Health care use, which is affected by the initiation of buprenorphine in people with OUD,^92,93^ was also not examined.

## Conclusions

This systematic literature review found that buprenorphine was associated with reduced chronic pain without precipitating opioid withdrawal or serious adverse effects in patients with chronic pain who used LTOT. The findings, although synthesized from studies that were of low to very low quality, suggested that buprenorphine rotation is a viable strategy for mitigating the harms of LTOT. Future research is warranted to address the optimal starting dose, formulation, and administration frequency of buprenorphine as well as the best approach to buprenorphine rotation.

## Notes

Supplement. **eAppendix.** Full Electronic Search Strategy **eFigure.** Population, Intervention, Comparator, Outcome, Timing, Setting (PICOTS) Question **eTable 1.** Full Study Details **eTable 2.** Details of GRADE Scores by Outcome **eTable 3.** Cochrane Risk of Bias Assessment Tool Results for Randomized Controlled Trials (RCTs) **eTable 4.** Newcastle Ottawa Scale Risk of Bias Assessment for Case Control Studies **eTable 5.** Newcastle Ottawa Scale Risk of Bias Assessment for Cohort Studies **eTable 6.** Details of Buprenorphine Rotation Protocols

## References

[1] Busse JW, Wang L, Kamaleldin M, . Opioids for chronic noncancer pain: a systematic review and meta-analysis. JAMA. 2018;320(23):2448-2460. doi:10.1001/jama.2018.18472 30561481 PMC6583638

[2] Chou R, Hartung D, Turner J, . *Opioid treatments for chronic pain*. Comparative Effectiveness Review No. 229. Pacific Northwest Evidence-Based Practice Center, Agency for Healthcare Research and Quality; 2020. AHRQ Publication No. 20-EHC011. doi:10.23970/AHRQEPCCER229 32338848

[3] Chou R, Turner JA, Devine EB, . The effectiveness and risks of long-term opioid therapy for chronic pain: a systematic review for a National Institutes of Health Pathways to Prevention Workshop. Ann Intern Med. 2015;162(4):276-286. doi:10.7326/M14-2559 25581257

[4] Els C, Jackson TD, Kunyk D, . Adverse events associated with medium- and long-term use of opioids for chronic non-cancer pain: an overview of Cochrane Reviews. Cochrane Database Syst Rev. 2017;10(10):CD012509. doi:10.1002/14651858.CD012509 29084357 PMC6485910

[5] Bohnert ASBB, Valenstein M, Bair MJ, . Association between opioid prescribing patterns and opioid overdose-related deaths. JAMA. 2011;305(13):1315-1321. doi:10.1001/jama.2011.370 21467284

[6] Dowell D, Haegerich TM, Chou R. CDC guideline for prescribing opioids for chronic pain—United States, 2016. MMWR Recomm Rep. 2016;65(1):1-49. doi:10.15585/mmwr.rr6501e1 26987082

[7] Frank JW, Lovejoy TI, Becker WC, . Patient outcomes in dose reduction or discontinuation of long-term opioid therapy: a systematic review. Ann Intern Med. 2017;167(3):181-191. doi:10.7326/M17-0598 28715848

[8] Chou R, Ballantyne J, Lembke A. Rethinking opioid dose tapering, prescription opioid dependence, and indications for buprenorphine. Ann Intern Med. 2019;171(6):427-429. doi:10.7326/M19-1488 31450240

[9] US Food and Drug Administration. FDA identifies harm reported from sudden discontinuation of opioid pain medicines and requires label changes to guide prescribers on gradual, individualized tapering. FDA Drug Safety Communication. April 9, 2019. Accessed October 12, 2020. https://www.fda.gov/drugs/drug-safety-and-availability/fda-identifies-harm-reported-sudden-discontinuation-opioid-pain-medicines-and-requires-label-changes

[10] Demidenko MI, Dobscha SK, Morasco BJ, Meath THA, Ilgen MA, Lovejoy TI. Suicidal ideation and suicidal self-directed violence following clinician-initiated prescription opioid discontinuation among long-term opioid users. Gen Hosp Psychiatry. 2017;47:29-35. doi:10.1016/j.genhosppsych.2017.04.011 28807135

[11] Webster L, Gudin J, Raffa RB, . Understanding buprenorphine for use in chronic pain: expert opinion. Pain Med. 2020;21(4):714-723. doi:10.1093/pm/pnz356 31917418 PMC7139205

[12] Dupouy J, Palmaro A, Fatséas M, . Mortality associated with time in and out of buprenorphine treatment in French office-based general practice: a 7-year cohort study. Ann Fam Med. 2017;15(4):355-358. doi:10.1370/afm.2098 28694272 PMC5505455

[13] Walsh SL, Preston KL, Stitzer ML, Cone EJ, Bigelow GE. Clinical pharmacology of buprenorphine: ceiling effects at high doses. Clin Pharmacol Ther. 1994;55(5):569-580. doi:10.1038/clpt.1994.71 8181201

[14] Dahan A, Yassen A, Romberg R, . Buprenorphine induces ceiling in respiratory depression but not in analgesia. Br J Anaesth. 2006;96(5):627-632. doi:10.1093/bja/ael051 16547090

[15] Sordo L, Barrio G, Bravo MJ, . Mortality risk during and after opioid substitution treatment: systematic review and meta-analysis of cohort studies. BMJ. 2017;357:j1550. doi:10.1136/bmj.j1550 28446428 PMC5421454

[16] Pergolizzi J, Böger RH, Budd K, . Opioids and the management of chronic severe pain in the elderly: consensus statement of an international expert panel with focus on the six clinically most often used World Health Organization step III opioids (buprenorphine, fentanyl, hydromorphone, methadone, morphine, oxycodone). Pain Pract. 2008;8(4):287-313. doi:10.1111/j.1533-2500.2008.00204.x 18503626

[17] Kress HG. Clinical update on the pharmacology, efficacy and safety of transdermal buprenorphine. Eur J Pain. 2009;13(3):219-230. doi:10.1016/j.ejpain.2008.04.011 18567516

[18] Hallinan R, Byrne A, Agho K, McMahon CG, Tynan P, Attia J. Hypogonadism in men receiving methadone and buprenorphine maintenance treatment. Int J Androl. 2009;32(2):131-139. doi:10.1111/j.1365-2605.2007.00824.x 17971165

[19] Yee A, Loh HS, Danaee M, Riahi S, Ng CG, Sulaiman AH. Plasma testosterone and sexual function in Southeast Asian men receiving methadone and buprenorphine maintenance treatment. J Sex Med. 2018;15(2):159-166. doi:10.1016/j.jsxm.2017.12.004 29275046

[20] Davis MP. Twelve reasons for considering buprenorphine as a frontline analgesic in the management of pain. J Support Oncol. 2012;10(6):209-219. doi:10.1016/j.suponc.2012.05.002 22809652

[21] Franchi S, Moschetti G, Amodeo G, Sacerdote P. Do all opioid drugs share the same immunomodulatory properties? A review from animal and human studies. Front Immunol. 2019;10:2914. doi:10.3389/fimmu.2019.02914 31921173 PMC6920107

[22] Wolff RF, Aune D, Truyers C, . Systematic review of efficacy and safety of buprenorphine versus fentanyl or morphine in patients with chronic moderate to severe pain. Curr Med Res Opin. 2012;28(5):833-845. doi:10.1185/03007995.2012.678938 22443154

[23] Filitz J, Griessinger N, Sittl R, Likar R, Schüttler J, Koppert W. Effects of intermittent hemodialysis on buprenorphine and norbuprenorphine plasma concentrations in chronic pain patients treated with transdermal buprenorphine. Eur J Pain. 2006;10(8):743-748. doi:10.1016/j.ejpain.2005.12.001 16426877

[24] Nasser AF, Heidbreder C, Liu Y, Fudala PJ. Pharmacokinetics of sublingual buprenorphine and naloxone in subjects with mild to severe hepatic impairment (Child-Pugh classes A, B, and C), in hepatitis C virus-seropositive subjects, and in healthy volunteers. Clin Pharmacokinet. 2015;54(8):837-849. doi:10.1007/s40262-015-0238-6 25603822

[25] Vadivelu N, Hines RL. Management of chronic pain in the elderly: focus on transdermal buprenorphine. Clin Interv Aging. 2008;3(3):421-430. doi:10.2147/CIA.S1880 18982913 PMC2682375

[26] Substance Abuse and Mental Health Services Administration. *Medications for Opioid Use Disorder. Treatment Improvement Protocol (TIP) Series 63*. Publication No. PEP20-02-01-006. Substance Abuse and Mental Health Services Administration; 2020.

[27] Webster L, Gruener D, Kirby T, Xiang Q, Tzanis E, Finn A. Evaluation of the tolerability of switching patients on chronic full μ-opioid agonist therapy to buccal buprenorphine. Pain Med. 2016;17(5):899-907. doi:10.1093/pm/pnv110 26917621 PMC4984426

[28] US Department of Health and Human Services, Office for Human Research Protections. Exemptions (2018 Requirements). 45 CFR §46.104 (2018). Accessed July 30, 2021. https://www.hhs.gov/ohrp/regulations-and-policy/regulations/45-cfr-46/common-rule-subpart-a-46104/index.html

[29] Volkow ND, McLellan AT. Opioid abuse in chronic pain–misconceptions and mitigation strategies. N Engl J Med. 2016;374(13):1253-1263. doi:10.1056/NEJMra1507771 27028915

[30] Boscarino JA, Hoffman SN, Han JJ. Opioid-use disorder among patients on long-term opioid therapy: impact of final DSM-5 diagnostic criteria on prevalence and correlates. Subst Abuse Rehabil. 2015;6:83-91. doi:10.2147/SAR.S85667 26316838 PMC4548725

[31] Hasin DS, O’Brien CP, Auriacombe M, . DSM-5 criteria for substance use disorders: recommendations and rationale. Am J Psychiatry. 2013;170(8):834-851. doi:10.1176/appi.ajp.2013.12060782 23903334 PMC3767415

[32] Sterne JAC, Savović J, Page MJ, . RoB 2: a revised tool for assessing risk of bias in randomised trials. BMJ. 2019;366:l4898. doi:10.1136/bmj.l4898 31462531

[33] Wells GA, Shea B, O’Connell D, . The Newcastle-Ottawa Scale (NOS) for assessing the quality of nonrandomized studies in meta-analyses. Accessed July 30, 2021. http://www.ohri.ca/programs/clinical_epidemiology/oxford.asp

[34] Guyatt GH, Oxman AD, Vist GE, ; GRADE Working Group. GRADE: an emerging consensus on rating quality of evidence and strength of recommendations. BMJ. 2008;336(7650):924-926. doi:10.1136/bmj.39489.470347.AD 18436948 PMC2335261

[35] Blondell RD, Ashrafioun L, Dambra CM, Foschio EM, Zielinski AL, Salcedo DM. A clinical trial comparing tapering doses of buprenorphine with steady doses for chronic pain and coexistent opioid addiction. J Addict Med. 2010;4(3):140-146. doi:10.1097/ADM.0b013e3181ba895d 20959867 PMC2931595

[36] Roux P, Sullivan MA, Cohen J, . Buprenorphine/naloxone as a promising therapeutic option for opioid abusing patients with chronic pain: reduction of pain, opioid withdrawal symptoms, and abuse liability of oral oxycodone. Pain. 2013;154(8):1442-1448. doi:10.1016/j.pain.2013.05.004 23707283 PMC3770461

[37] Neumann AM, Blondell RD, Hoopsick RA, Homish GG. Randomized clinical trial comparing buprenorphine/naloxone and methadone for the treatment of patients with failed back surgery syndrome and opioid addiction. J Addict Dis. 2020;38(1):33-41. doi:10.1080/10550887.2019.1690929 31774028 PMC7082187

[38] Weiss RD, Potter JS, Fiellin DA, . Adjunctive counseling during brief and extended buprenorphine-naloxone treatment for prescription opioid dependence: a 2-phase randomized controlled trial. Arch Gen Psychiatry. 2011;68(12):1238-1246. doi:10.1001/archgenpsychiatry.2011.121 22065255 PMC3470422

[39] Baron MJ, McDonald PW. Significant pain reduction in chronic pain patients after detoxification from high-dose opioids. J Opioid Manag. 2006;2(5):277-282. doi:10.5055/jom.2006.0041 17319259

[40] Sturgeon JA, Sullivan MD, Parker-Shames S, Tauben D, Coelho P. Outcomes in long-term opioid tapering and buprenorphine transition: a retrospective clinical data analysis. Pain Med. 2020;21(12):3635-3644. doi:10.1093/pm/pnaa029 32163149

[41] Nielsen S, Hillhouse M, Weiss RD, . The relationship between primary prescription opioid and buprenorphine-naloxone induction outcomes in a prescription opioid dependent sample. Am J Addict. 2014;23(4):343-348. doi:10.1111/j.1521-0391.2013.12105.x 24112096 PMC4151625

[42] Worley MJ, Heinzerling KG, Shoptaw S, Ling W. Pain volatility and prescription opioid addiction treatment outcomes in patients with chronic pain. Exp Clin Psychopharmacol. 2015;23(6):428-435. doi:10.1037/pha0000039 26302337 PMC4658240

[43] Worley MJ, Heinzerling KG, Shoptaw S, Ling W. Volatility and change in chronic pain severity predict outcomes of treatment for prescription opioid addiction. Addiction. 2017;112(7):1202-1209. doi:10.1111/add.13782 28164407 PMC5461207

[44] Griffin ML, McDermott KA, McHugh RK, Fitzmaurice GM, Jamison RN, Weiss RD. Longitudinal association between pain severity and subsequent opioid use in prescription opioid dependent patients with chronic pain. Drug Alcohol Depend. 2016;163:216-221. doi:10.1016/j.drugalcdep.2016.04.023 27161860 PMC4880512

[45] Weiss RD, Griffin ML, Potter JS, . Who benefits from additional drug counseling among prescription opioid-dependent patients receiving buprenorphine-naloxone and standard medical management? Drug Alcohol Depend. 2014;140:118-122. doi:10.1016/j.drugalcdep.2014.04.005 24831754 PMC4053488

[46] Aurilio C, Pace MC, Pota V, . Opioids switching with transdermal systems in chronic cancer pain. J Exp Clin Cancer Res. 2009;28(1):61. doi:10.1186/1756-9966-28-61 19422676 PMC2684533

[47] Berland DW, Malinoff HL, Weiner MA, Przybylski R. When opioids fail in chronic pain management: the role for buprenorphine and hospitalization. Am J Ther. 2013;20(4):316-321. doi:10.1097/MJT.0b013e31827ab599 23584313

[48] Pade PA, Cardon KE, Hoffman RM, Geppert CMA. Prescription opioid abuse, chronic pain, and primary care: a co-occurring disorders clinic in the chronic disease model. J Subst Abuse Treat. 2012;43(4):446-450. doi:10.1016/j.jsat.2012.08.010 22980449

[49] Daitch J, Frey ME, Silver D, Mitnick C, Daitch D, Pergolizzi J Jr. Conversion of chronic pain patients from full-opioid agonists to sublingual buprenorphine. Pain Physician. 2012;15(3 suppl):ES59-ES66. doi:10.36076/ppj.2012/15/ES59 22786462

[50] Daitch D, Daitch J, Novinson D, Frey M, Mitnick C, Pergolizzi J Jr. Conversion from high-dose full-opioid agonists to sublingual buprenorphine reduces pain scores and improves quality of life for chronic pain patients. Pain Med. 2014;15(12):2087-2094. doi:10.1111/pme.12520 25220043

[51] Freye E, Anderson-Hillemacher A, Ritzdorf I, Levy JV. Opioid rotation from high-dose morphine to transdermal buprenorphine (Transtec) in chronic pain patients. Pain Pract. 2007;7(2):123-129. doi:10.1111/j.1533-2500.2007.00119.x 17559481

[52] Malinoff HL, Barkin RL, Wilson G. Sublingual buprenorphine is effective in the treatment of chronic pain syndrome. Am J Ther. 2005;12(5):379-384. doi:10.1097/01.mjt.0000160935.62883.ff 16148422

[53] Tang VM, Lam-Shang-Leen J, Brothers TD, . Case series: limited opioid withdrawal with use of transdermal buprenorphine to bridge to sublingual buprenorphine in hospitalized patients. Am J Addict. 2020;29(1):73-76. doi:10.1111/ajad.12964 31626394 PMC7134509

[54] Rosenblum A, Cruciani RA, Strain EC, . Sublingual buprenorphine/naloxone for chronic pain in at-risk patients: development and pilot test of a clinical protocol. J Opioid Manag. 2012;8(6):369-382. doi:10.5055/jom.2012.0137 23264315 PMC3630795

[55] Streltzer J, Davidson R, Goebert D. An observational study of buprenorphine treatment of the prescription opioid dependent pain patient. Am J Addict. 2015;24(4):357-361. doi:10.1111/ajad.12198 25675861

[56] Weiss RD, Potter JS, Provost SE, . A multi-site, two-phase, Prescription Opioid Addiction Treatment Study (POATS): rationale, design, and methodology. Contemp Clin Trials. 2010;31(2):189-199. doi:10.1016/j.cct.2010.01.003 20116457 PMC2831115

[57] Wesson DR, Ling W. The Clinical Opiate Withdrawal Scale (COWS). J Psychoactive Drugs. 2003;35(2):253-259. doi:10.1080/02791072.2003.10400007 12924748

[58] Handelsman L, Cochrane KJ, Aronson MJ, Ness R, Rubinstein KJ, Kanof PD. Two new rating scales for opiate withdrawal. Am J Drug Alcohol Abuse. 1987;13(3):293-308. doi:10.3109/00952998709001515 3687892

[59] Pergolizzi JV Jr, Raffa RB. Safety and efficacy of the unique opioid buprenorphine for the treatment of chronic pain. J Pain Res. 2019;12:3299-3317. doi:10.2147/JPR.S231948 31997882 PMC6917545

[60] Hale M, Urdaneta V, Kirby MT, Xiang Q, Rauck R. Long-term safety and analgesic efficacy of buprenorphine buccal film in patients with moderate-to-severe chronic pain requiring around-the-clock opioids. J Pain Res. 2017;10:233-240. doi:10.2147/JPR.S120170 28182123 PMC5279817

[61] Schuster M, Bayer O, Heid F, Laufenberg-Feldmann R. Opioid rotation in cancer pain treatment. Dtsch Arztebl Int. 2018;115(9):135-142. doi:10.3238/arztebl.2018.0135 29563006 PMC5876542

[62] Knotkova H, Fine PG, Portenoy RK. Opioid rotation: the science and the limitations of the equianalgesic dose table. J Pain Symptom Manage. 2009;38(3):426-439. doi:10.1016/j.jpainsymman.2009.06.001 19735903

[63] Mercadante S, Bruera E. Opioid switching in cancer pain: from the beginning to nowadays. Crit Rev Oncol Hematol. 2016;99:241-248. doi:10.1016/j.critrevonc.2015.12.011 26806145

[64] Elkader A, Sproule B. Buprenorphine: clinical pharmacokinetics in the treatment of opioid dependence. Clin Pharmacokinet. 2005;44(7):661-680. doi:10.2165/00003088-200544070-00001 15966752

[65] McCormack K. Signal transduction in neuropathic pain, with special emphasis on the analgesic role of opioids—part II: moving basic science towards a new pharmacotherapy. Pain Rev. 1999;6(2):99-131. doi:10.1191/096813099669853099

[66] Koppert W, Ihmsen H, Körber N, . Different profiles of buprenorphine-induced analgesia and antihyperalgesia in a human pain model. Pain. 2005;118(1-2):15-22. doi:10.1016/j.pain.2005.06.030 16154698

[67] Sánchez-Blázquez P, Gómez-Serranillos P, Garzón J. Agonists determine the pattern of G-protein activation in μ-opioid receptor-mediated supraspinal analgesia. Brain Res Bull. 2001;54(2):229-235. doi:10.1016/S0361-9230(00)00448-2 11275413

[68] Lutfy K, Eitan S, Bryant CD, . Buprenorphine-induced antinociception is mediated by mu-opioid receptors and compromised by concomitant activation of opioid receptor-like receptors. J Neurosci. 2003;23(32):10331-10337. doi:10.1523/JNEUROSCI.23-32-10331.2003 14614092 PMC6741014

[69] Fiellin DA, Schottenfeld RS, Cutter CJ, Moore BA, Barry DT, O’Connor PG. Primary care-based buprenorphine taper vs maintenance therapy for prescription opioid dependence: a randomized clinical trial. JAMA Intern Med. 2014;174(12):1947-1954. doi:10.1001/jamainternmed.2014.5302 25330017 PMC6167926

[70] Ling W, Hillhouse M, Domier C, . Buprenorphine tapering schedule and illicit opioid use. Addiction. 2009;104(2):256-265. doi:10.1111/j.1360-0443.2008.02455.x 19149822 PMC3150159

[71] Bentzley BS, Barth KS, Back SE, Book SW. Discontinuation of buprenorphine maintenance therapy: perspectives and outcomes. J Subst Abuse Treat. 2015;52:48-57. doi:10.1016/j.jsat.2014.12.011 25601365 PMC4382404

[72] Ballantyne JC, Sullivan MD, Koob GF. Refractory dependence on opioid analgesics. Pain. 2019;160(12):2655-2660. doi:10.1097/j.pain.0000000000001680 31408053

[73] Koob GF. Neurobiology of opioid addiction: opponent process, hyperkatifeia, and negative reinforcement. Biol Psychiatry. 2020;87(1):44-53. doi:10.1016/j.biopsych.2019.05.023 31400808

[74] Hämmig R, Kemter A, Strasser J, . Use of microdoses for induction of buprenorphine treatment with overlapping full opioid agonist use: the Bernese method. Subst Abuse Rehabil. 2016;7:99-105. doi:10.2147/SAR.S109919 27499655 PMC4959756

[75] Ahmed S, Bhivandkar S, Lonergan BB, Suzuki J. Microinduction of buprenorphine/naloxone: a review of the literature. Am J Addict. 2021;30(4):305-315. doi:10.1111/ajad.13135 33378137

[76] Lee DS, Hann JE, Klaire SS, Nikoo M, Negraeff MD, Rezazadeh-Azar P. Rapid induction of buprenorphine/naloxone for chronic pain using a microdosing regimen: a case report. A A Pract. 2020;14(2):44-47. doi:10.1213/XAA.0000000000001138 31770128 PMC7147949

[77] Terasaki D, Smith C, Calcaterra SL. Transitioning hospitalized patients with opioid use disorder from methadone to buprenorphine without a period of opioid abstinence using a microdosing protocol. Pharmacotherapy. 2019;39(10):1023-1029. doi:10.1002/phar.2313 31348544

[78] Weimer MB, Guerra M, Morrow G, Adams K. Hospital-based buprenorphine micro-dose initiation. J Addict Med. 2021;15(3):255-257. doi:10.1097/ADM.0000000000000745 32960820

[79] Mercadante S, Casuccio A, Tirelli W, Giarratano A. Equipotent doses to switch from high doses of opioids to transdermal buprenorphine. Support Care Cancer. 2009;17(6):715-718. doi:10.1007/s00520-008-0546-6 19104845

[80] Sittl R, Likar R, Nautrup BP. Equipotent doses of transdermal fentanyl and transdermal buprenorphine in patients with cancer and noncancer pain: results of a retrospective cohort study. Clin Ther. 2005;27(2):225-237. doi:10.1016/j.clinthera.2005.02.012 15811486

[81] Hans G, Robert D. Transdermal buprenorphine—a critical appraisal of its role in pain management. J Pain Res. 2009;2:117-134. doi:10.2147/JPR.S6503 21197300 PMC3004620

[82] Skaer TL. Dosing considerations with transdermal formulations of fentanyl and buprenorphine for the treatment of cancer pain. J Pain Res. 2014;7:495-503. doi:10.2147/JPR.S36446 25170278 PMC4145844

[83] Mendelson J, Upton RA, Everhart ET, Jacob P III, Jones RT. Bioavailability of sublingual buprenorphine. J Clin Pharmacol. 1997;37(1):31-37. doi:10.1177/009127009703700106 9048270

[84] Vowles KE, McEntee ML, Julnes PS, Frohe T, Ney JP, van der Goes DN. Rates of opioid misuse, abuse, and addiction in chronic pain: a systematic review and data synthesis. Pain. 2015;156(4):569-576. doi:10.1097/01.j.pain.0000460357.01998.f1 25785523

[85] Ballantyne JC, Sullivan MD, Kolodny A. Opioid dependence vs addiction: a distinction without a difference? Arch Intern Med. 2012;172(17):1342-1343. doi:10.1001/archinternmed.2012.3212 22892799

[86] Ballantyne JC. Assessing the prevalence of opioid misuse, abuse, and addiction in chronic pain. Pain. 2015;156(4):567-568. doi:10.1097/j.pain.0000000000000105 25630030

[87] Manhapra A, Arias AJ, Ballantyne JC. The conundrum of opioid tapering in long-term opioid therapy for chronic pain: a commentary. Subst Abus. 2018;39(2):152-161. doi:10.1080/08897077.2017.1381663 28929914 PMC6129223

[88] Dworkin RH, Turk DC, Wyrwich KW, . Interpreting the clinical importance of treatment outcomes in chronic pain clinical trials: IMMPACT recommendations. J Pain. 2008;9(2):105-121. doi:10.1016/j.jpain.2007.09.005 18055266

[89] Salas J, Scherrer JF, Schneider FD, . New-onset depression following stable, slow, and rapid rate of prescription opioid dose escalation. Pain. 2017;158(2):306-312. doi:10.1097/j.pain.0000000000000763 28092649 PMC7050294

[90] Scherrer JF, Svrakic DM, Freedland KE, . Prescription opioid analgesics increase the risk of depression. J Gen Intern Med. 2014;29(3):491-499. doi:10.1007/s11606-013-2648-1 24165926 PMC3930792

[91] Robertson JA, Purple RJ, Cole P, Zaiwalla Z, Wulff K, Pattinson KTS. Sleep disturbance in patients taking opioid medication for chronic back pain. Anaesthesia. 2016;71(11):1296-1307. doi:10.1111/anae.13601 27545291 PMC5082544

[92] Baser O, Chalk M, Fiellin DA, Gastfriend DR. Cost and utilization outcomes of opioid-dependence treatments. Am J Manag Care. 2011;17(suppl 8):S235-S248. 21761950

[93] Ronquest NA, Willson TM, Montejano LB, Nadipelli VR, Wollschlaeger BA. Relationship between buprenorphine adherence and relapse, health care utilization and costs in privately and publicly insured patients with opioid use disorder. Subst Abuse Rehabil. 2018;9:59-78. doi:10.2147/SAR.S150253 30310349 PMC6165853

