# Supplementary material for: Evaluation of Buprenorphine Rotation in Patients Receiving Long-term Opioids for Chronic Pain: A Systematic Review
Source: JAMA Netw Open. 2021 Sep 8;4(9):e2124152. doi: 10.1001/jamanetworkopen.2021.24152 (PMC8427372; doi:10.1001/jamanetworkopen.2021.24152)
Supplement: Supplement. — eAppendix. Full Electronic Search Strategy eFigure. Population, Intervention, Comparator, Outcome, Timing, Setting (PICOTS) Question eTable 1. Full Study Details eTable 2. Details of GRADE Scores by Outcome eTable 3. Cochrane Risk of Bias Assessment Tool Results for Randomized Controlled Trials (RCTs) eTable 4. Newcastle Ottawa Scale Risk of Bias Assessment for Case Control Studies eTable 5. Newcastle Ottawa Scale Risk of Bias Assessment for Cohort Studies eTable 6. Details of Buprenorphine Rotation Protocols [file jamanetwopen-e2124152-s001.pdf]

Supplementary Online Content

Powell VD, Rosenberg JM, Yaganti A, et al. Evaluation of buprenorphine rotation in patients receiving long-term opioids for chronic pain: a systematic review. *JAMA Netw Open*. 2021;4(9):e2124152. doi:10.1001/jamanetworkopen.2021.24152

eAppendix. Full Electronic Search Strategy

eFigure. Population, Intervention, Comparator, Outcome, Timing, Setting (PICOTS) Question

eTable 1. Full Study Details

eTable 2. Details of GRADE Scores by Outcome

eTable 3. Cochrane Risk of Bias Assessment Tool Results for Randomized Controlled Trials (RCTs)

eTable 4. Newcastle Ottawa Scale Risk of Bias Assessment for Case Control Studies

eTable 5. Newcastle Ottawa Scale Risk of Bias Assessment for Cohort Studies

eTable 6. Details of Buprenorphine Rotation Protocols

This supplementary material has been provided by the authors to give readers additional information about their work.

© 2021 Powell VD et al. *JAMA Network Open.*

eAppendix. Full Electronic Search Strategy

PubMed Search 29 March 2020

((((((((((Chronic pain[mh] OR chronic pain[tiab] OR opioid related disorders[mh] OR opioid related disorder*[tiab])))) OR ((((Analgesics, Opioid[MeSH Terms]) OR ((opiate*[Title/Abstract] OR opioid*[Title/Abstract] OR fentanyl[Title/Abstract] OR narcotic*[Title/Abstract] OR dilaudid[Title/Abstract] OR oxycontin*[Title/Abstract] OR oxycod*[Title/Abstract]))))) OR ((Drug Prescriptions[MeSH Terms]) OR ((prescript*[Title/Abstract] OR prescrib*[Title/Abstract] OR pharmaceutical*[Title/Abstract] OR legal*[Title/Abstract]))))))
AND
((buprenorphine[mh] OR buprenorphine[tiab] OR opioid agonist therapies[tiab] OR Opiate Substitution Treatment[mh] OR opioid use disorder[tiab])))
AND
((treatment outcome[mh] OR treatment outcome*[tiab] OR pain management[tiab] OR pain control[tiab] OR pain[majr] OR quality of life[mh] OR quality of life[tiab]))))))
N = 1307
Filters: English; Adult: 19+ years N = 915 + 57 unindexed articles

Search rerun 29 Oct 2020 N = 10

Embase searches 23 April 2020

1. 'chronic pain'/exp OR 'chronic pain':ti,ab OR 'opioid related disorders'/exp OR 'opioid related disorder*':ti,ab N = 94,738
2. 'analgesics, opioid'/exp OR opiate*:ti,ab OR opioid*:ti,ab OR fentanyl:ti,ab OR narcotic*:ti,ab OR dilaudid:ti,ab OR oxycontin*:ti,ab OR oxycod*:ti,ab N = 405,820
3. 'drug prescriptions'/exp OR prescript*:ti,ab OR prescrib*:ti,ab OR pharmaceutical*:ti,ab OR legal*:ti,ab N = 703,856
4. #2 AND #3 N = 38,349
5. #1 OR #4 N = 123,765
6. 'buprenorphine'/exp OR buprenorphine:ti,ab OR 'opioid agonist therapies':ti,ab OR 'opiate substitution treatment'/exp OR 'opioid use disorder':ti,ab N = 21,471
7. 'treatment outcome'/exp OR 'treatment outcome*':ti,ab OR 'pain management':ti,ab OR 'pain control':ti,ab OR 'pain'/exp/mj OR 'quality of life'/exp OR 'quality of life':ti,ab OR 'treatment response'/exp N = 2,684,007
8. #5 AND #6 AND #7 N = 3516
9. #8 AND [review]/lim N = 870
10. #8 NOT #9 N = 2646
11. #8 NOT #9 AND [english]/lim AND ([adult]/lim OR [aged]/lim) N = 1442

Searches rerun 29 October 2020) N = 178

CINAHL searches 24 April 2020
((((((((((MH "Chronic pain+") OR TI "chronic pain" OR AB "chronic pain" OR (MH "opioid related disorders+") OR TI "opioid related disorder*" OR AB "opioid related disorder*")))) OR (((((MH "Analgesics, Opioid+")) OR ((TI opiate* OR AB opiate* OR TI opioid* OR AB opioid* OR TI fentanyl OR AB fentanyl OR TI narcotic* OR AB narcotic* OR TI dilaudid OR AB dilaudid OR TI oxycontin* OR AB oxycontin* OR TI oxycod* OR AB oxycod*)))) AND (((MH "Drug Prescriptions+")) OR ((TI prescript* OR AB prescript* OR TI prescrib* OR AB prescrib* OR TI pharmaceutical* OR AB pharmaceutical* OR TI legal* OR AB legal*)))))) AND (((MH "buprenorphine+") OR TI buprenorphine OR AB buprenorphine OR TI "opioid agonist therapies" OR AB "opioid agonist therapies" OR (MH "Opiate Substitution Treatment+") OR TI "opioid use disorder" OR AB "opioid use disorder"))) AND (((MH "treatment outcome+") OR TI "treatment outcome*" OR AB "treatment outcome*" OR TI "pain management" OR AB "pain management" OR TI "pain control" OR AB "pain control" OR (MM "pain+") OR (MH "quality of life+") OR TI "quality of life" OR AB "quality of life"))))) N = 421
Filters: Academic Journals, Age: - all adult and Language: - english N = 175
PT Review N = 46

© 2021 Powell VD et al. *JAMA Network Open.*

Final N =131

Searches rerun 3 Nov 202) N = 0

PsycINFO Search 24 April 2020

((((((((("Chronic pain" OR "chronic pain".ti,ab OR "opioid related disorders" OR "opioid related disorder*".ti,ab)))) OR (((("Analgesics, Opioid") OR ((opiate*.ti,ab OR opioid*.ti,ab OR fentanyl.ti,ab OR narcotic*.ti,ab OR dilaudid.ti,ab OR oxycontin*.ti,ab OR oxycod*.ti,ab)))) AND (("Drug Prescriptions") OR ((prescript*.ti,ab OR prescrib*.ti,ab OR pharmaceutical*.ti,ab OR legal*.ti,ab)))))) AND ((buprenorphine OR buprenorphine.ti,ab OR "opioid agonist therapies".ti,ab OR "Opiate Substitution Treatment" OR "opioid use disorder".ti,ab))) AND (("treatment outcome" OR "treatment outcome*".ti,ab OR "pain management".ti,ab OR "pain control".ti,ab OR exp *pain/ OR "quality of life" OR "quality of life".ti,ab)))) N = 408
Filters: Peer-reviewed, Age: adulthood (18 yrs & older), Language: English,Review N = 323

Search rerun 3 Nov 2020 N = 3

© 2021 Powell VD et al. *JAMA Network Open.*

eFigure. Population, Intervention, Comparator, Outcome, Timing, Setting (PICOTS) Question

Population: Adult patients with chronic pain on long-term opioid therapy, with or without a diagnosis of opioid use disorder (OUD).

Intervention: Transition to buprenorphine from any prescribed full mu-opioid receptor (MOR) agonist(s) including: transdermal fentanyl, oxycodone, methadone, morphine, meperidine, hydrocodone, oxymorphone, hydromorphone, tramadol, tapentadol, codeine.

Comparator: Any or none

Outcomes:

1. Precipitated opioid withdrawal symptoms
2. Pain (intensity and interference with functioning)
3. Completion of protocol / continuation of buprenorphine protocol (ie., success of rotation)
4. Adverse events and side effects
5. Mental health (ie., depressive symptoms, anxiety symptoms, sleep quality, etc.)
6. Health care utilization (ie., emergency department visits, hospitalizations, etc.)

Time: Variable, will not exclude articles based on length of study or outcome assessment

Study Type: Observational study (controlled or uncontrolled) or randomized, controlled trial (excludes reviews, case reports, gray literature)

© 2021 Powell VD et al. *JAMA Network Open.*

eTable 1. Full Study Details

Study	Population/ Participants	Study Design and Groups	Buprenorphine Induction and Titration Details	Measure(s)	Outcome(s)	Risk of Bias (ROB) Assessment
Weiss et al, 2011.	The prescription opioid addiction treatment study (POATS) enrolled n = 653 total, with n = 274 (42%) reporting chronic pain. All participants with self-identified dependence on prescription opioids.<br><br>Most with chronic pain reported it for ≥1 year (94%), with 52% reporting pain for ≥4 years. Mean duration of opioid use 5.2 years (SD=4.7), and reported physical pain relief as primary reason for initial use (82%).<br><br>About 23% of participants had ever used heroin, but using heroin as the primary opioid was an exclusion criterion.<br><br>Date of study: June 2006-July 2009	2-phase, adaptive treatment, randomized controlled trial (RCT).<br><br>This is the original POATS study publication reporting outcomes of primary analysis.<br><br>Participants randomized to standard medical management or extended opioid-dependence counseling in each phase.<br><br>Phase 1: participants received buprenorphine for two weeks, then tapered over two weeks, and followed for 8 weeks.<br><br>Phase 2: Individuals who failed phase 1 (ie., returned to opioid use) received 12 weeks of steady-dose buprenorphine, a 4-week taper, and 8 week follow-up period.	Participants instructed to stop short-acting opioids ≥12 hours prior to induction. Those previously on methadone (≤40mg) were instructed to stop 36 hours prior.<br><br>When Clinical Opiate Withdrawal Scale (COWS) score was ≥8, they received 4 - 12 mg (in 4mg increments) on induction day.<br><br>At each subsequent visit, study physician could adjust the dose up to 8mg/week depending on withdrawal symptoms and adverse effects, but not for pain (range 8 - 32 mg/day).<br><br>During Phase 2 stabilization, the mean maximum dose was 20.3mg (SD=7.9, range 8-32mg/day) in a single daily dose.	Success in Phase 1 was measured by completing week 12 with ≤4 days/month of self-reported opioid use, absence of 2 consecutive positive urine drug screens, no substance use disorder(SUD) treatment other than self-help, and ≤1 missing urine sample.<br><br>Phase 2 success was defined as abstaining from opioids during the final week of buprenorphine maintenance and ≥2 of the 3 previous weeks.<br><br>Adverse Event (AE) reporting	Phase 1: Only 6.6% total with treatment success; success rate in chronic pain was 7.9% (p=0.25).<br><br>Phase 2: 49.2% with success while still taking buprenorphine; success rate in chronic pain was 53% (p=0.25).<br><br>Participants most likely to reduce opioid use during buprenorphine maintenance than after tapering (49.2% vs. 8.6%, p<0.001).<br><br>Adding opioid dependence counseling did not improve outcome.<br><br>In Phase 2, participants much more likely to use additional opioids after tapering than while receiving buprenorphine OR 10.6 (95% CI 7.2-15.6)<br><br>Rate of opioid-positive urine tests in phase 2 was significantly higher during taper and	Some concerns<br><br>(Cochrane tool)

© 2021 Powell VD et al. *JAMA Network Open.*

					posttaper than while maintained on buprenorphine (58.1% vs. 39.1%, p<0.001).<br><br>Phase 1: Most (n=542, 83%) experienced ≥1 AEs, most common were headache (29%), constipation (16%), insomnia (13%). N=15 (2%) discontinued treatment due to AEs.<br><br>Phase 2: Most (n=216, 60%) experienced ≥1 AEs, most common were headache (27%), nasopharyngitis (24%), nausea (17%). N=9 (2.5%) discontinued buprenorphine due to adverse events.<br><br>12 serious AEs in Phase 1, 24 in Phase 2 (in 21 unique patients)<br><br>Depression leading to hospitalization occurred in n=5, all after completing buprenorphine taper.<br><br>Follow up: 20 weeks total for Phase 2 (12 week buprenorphine maintenance, 4 week taper, 8 week follow-up).	

© 2021 Powell VD et al. *JAMA Network Open.*

Neumann et al, 2020.	Participants (n=19) all with post-surgical chronic back pain and self-identified addiction to opioids prescribed to them. Most (n=13) women; most (n=17) employed.<br><br>Date of study: January 2012 - May 2014	Open-label randomized trial comparing methadone and SL buprenorphine/naloxone. Participants allowed to switch groups.	Starting dose range of 8 - 16 mg/day, dosed 2-4 times/daily.	Primary outcome: analgesia (Numeric Rating Scale (NRS), Visual Analog Scale (VAS), Brief Pain Inventory).<br><br>Secondary outcomes: Self-reported functioning (VAS, Roland Morris Disability Questionnaire); percent of participants completing 6 months of treatment; depression (Beck Depression Inventory(BDI-II))<br><br>Reported side effects at 6 months and ongoing safety/AE monitoring	10/19 (53%) completed study.<br><br>Groups differed at baseline; buprenorphine group with less pain ($p \leq 0.05$) and more alcohol use ($p=0.027$).<br><br>Both groups with average 20% improvement in pain via VAS ($p=0.002$) and BPI ($p=0.016$).<br><br>Both groups with average 21% improvement in function ($p=0.002$).<br><br>When buprenorphine was examined alone, there was no significant reduction in pain.<br><br>Both groups reported less severe depression at 6 months (mean BDI score 21.2 to 16.2) changing from moderate to mild depression ($p=0.01$).<br><br>Study enrollment was discontinued early due to two individuals (both in methadone group) abusing study drug.	High ROB<br><br>(Cochrane tool)

© 2021 Powell VD et al. *JAMA Network Open.*

					At 6 months, two participants each reported nausea/vomiting and sedation.sleepiness; n=4 constipation, n=1 craving, n=3 insomnia, n=3 headaches. (These results not reported by methadone or buprenorphine treatment condition.)<br><br>Follow up: 6 months	
Roux et al, 2013.	n=51 with mild - moderate chronic pain who met criteria for prescription opioid dependence (but were not treatment-seeking). Median duration of prescription opioid use was 5 years (IQR 2-8 years). Opioid dose median 60 oral morphine equivalent (OME) (IQR 38-144). Mean age 48 (range 43-54). N=9 female; n=5 employed.	RCT<br><br>Within-subjects, repeated-measures design.<br>Aimed to evaluate whether buprenorphine/naloxone at different doses would reduce willingness to self-administer varying doses of oxycodone vs receive money during laboratory sessions.<br><br>Multivariate models used to identify factors associated with oxycodone preference using first MPQ score and then Subjective Opiate Withdrawal Scale (SOWS) score as explanatory variables.	Starting dose 2, 8, or 16mg; each participant received all doses in random order under double blind conditions. There were no medication adjustments. Dosed four times/day.	Primary outcome was willingness to self-administer oxycodone under influence of buprenorphine.<br><br>McGill Pain Questionnaire (MPQ) given at baseline and during each of the buprenorphine dose conditions.<br><br>SOWS measured one hour after first daily dose of buprenorphine.<br><br>Measured completion of protocol rates and reasons for dropping out,	Mean MPQ one hour after buprenorphine 21 (range 15-31).<br><br>MPQ scores significantly decreased under buprenorphine maintenance (p<0.001); effect was dose-dependent as 8mg and 16mg doses decreased pain more than 2mg (p=0.03 and p<0.001, respectively);<br><br>Mean SOWS 1 hr post buprenorphine 4 (range 1-9). Adjusted OR for withdrawal symptoms under 16mg vs 2mg buprenorphine 0.41 (95% CI 0.20 – 0.82).<br><br>Higher SOWS scores with increased odds of	High ROB<br><br>(Cochrane tool)

© 2021 Powell VD et al. *JAMA Network Open.*

				including adverse events	oxycodone self-administration (Adjusted OR 1.96 (95% CI 1.29-3.00, p=0.002)<br><br>Those who reported more withdrawal symptoms and pain under any buprenorphine condition more likely to prefer oxycodone.<br><br>Of n=43 who entered inpatient study,18 did not complete and n=25 did. Reasons were intolerance to buprenorphine in n=3 (nausea and sedation). N=2 had psychiatric issuers, n=2 could not tolerate environment; n=5 with behavioral issues, n=5 lost to follow up; n=1 seeking treatment.<br><br>Follow up: Maintained on each dose of buprenorphine for two weeks only. No additional follow-up.	
Webster et al, 2016.	n=39 with chronic pain receiving around-the-clock opioid therapy (morphine or oxycodone) for at least 4 months (via personal communication with author) and confirmed	RCT<br><br>Aimed to determine whether opioid-dependent patients would experience withdrawal with rotation	Those using 80-160 OME (n=33) received 300µg buccal buprenorphine q12 hours; those using 161-220 OME (n=6) received 450µg q12 hours.	COWS, measured 0.5 hours before, and 0.5, 1, 1.5, 2, 2.5, 3, 3.5, 4, 6, 9, and 12 hours after first dose of study drug, and 0.5, 1,	Mean change in COWS from baseline (0.5 hours before buprenorphine) was similar for both groups.	High ROB<br><br>(Cochrane tool)

© 2021 Powell VD et al. *JAMA Network Open.*

	opioid-dependent by naloxone challenge. Dose range 80-220 OME/day. Mean age group 1=42 years; group 2=46 years. N=21 women.	to buprenorphine compared to a 50% dose reduction of current opioid.<br><br>Crossover design; all participants received both treatments in random order.	Doses were given 8-12 hours after last dose of full MOR agonist (ie., home medication).<br><br>Patients were admitted twice for two consecutive nights, 7-14 days apart. All received both buprenorphine and dose-reduced full MOR agonist but in random order.<br><br>Procedure: administered drug and observed for signs/symptoms of withdrawal for 12 hours, at which time a second dose of study drug was given, then monitored for another 12 hours. On day 3 (24 hours after receiving study drug), they were resumed on usual dose of full MOR agonist.	1.5, 2, 4, and 12 hours after the second dose.<br><br>Primary endpoint was proportion of patients who experienced opioid withdrawal (COWS ≥13, or required rescue medications over the 24 hour study period)<br><br>Pain changes before (0.5 hours) and after first dose (0.5, 1, 2, 4, 9, 12) and second dose (0.5, 1, 2, 4, and 12) via NRS (0-10).<br><br>Measured rates of completion of protocol.<br><br>Adverse event (standard safety) monitoring	n=2 experienced withdrawal (COWS≥13); one with both buprenorphine and dose-reduced full agonist and the other with dose -reduced full agonist only.<br><br>In both groups, COWS score peaked at 12 hours post dose, and decreased after second dose (no significant difference between dose-reduced full agonist or buprenorphine.)<br><br>For both buprenorphine and dose-reduced full agonist, pain increased up to 12 hours post first dose and then decreased after second dose. No significant difference between buprenorphine and full agonist (dose-reduced). No statistics given, but confidence intervals overlap on graph.<br><br>31/33 (94%) in Group 1 completed both study periods<br>5/6 (83%) in Group 2 completed both study periods.	

© 2021 Powell VD et al. *JAMA Network Open.*

					n=1 dropped out due to adverse event; n=2 lost to follow up.<br><br>Group 1: mean maximum COWS score over 24 hours was non-significantly lower than on dose-reduced full agonist (4.6 (SD=3.15) vs 5.3 (SD=4.4), p=0.79).<br><br>Group 2: Mean max COWS score non-significantly lower on buprenorphine (5.5(SD=1.9) vs 6.3(2.5), p=0.62).<br><br>AEs:<br>In Group 1, 18/32 (56%) and in Group 2, 1/6 (17%) with ≥1 treatment emergent adverse event (TEAE) under buprenorphine condition.<br><br>N=10 (31%) with AEs under buprenorphine condition including headache (19%), vomiting (13%), nausea, diarrhea, and drug withdrawal syndrome.<br><br>AEs:	

					Most common:<br>(Group 1)<br>GI: 22% (vomiting 13%)<br><br>General disorders/administration site conditions: 13%<br><br>Nervous system disorders: 25% (headache 19%); the rest occurring in <10%<br><br>Group 2: Single participant reported General disorders and administration site conditions (17%) and drug withdrawal syndrome (17%).<br><br>Follow up: received two doses only.	
Blondell et al, 2010.	Participants (n=12) had chronic, nonmalignant pain associated with self-identified prescription opioid addiction and were seeking treatment. Age range 24-65 years. N=6 (50%) women. N=8 not working.<br><br>Date of study: December 2007 - April 2008	RCT<br><br>Open-label; all admitted for stabilization with buprenorphine; on discharge they were randomized to receive buprenorphine taper (over 4 months) or steady dose. All encouraged to engage in behavioral and other pain treatments (counseling, pain psychologist, PMR, PCP)	Prior opioids stopped after midnight prior to admission date. Participants started on 4mg, then allowed additional 2mg every two hours until opioid withdrawal symptoms controlled. For those in the steady dose condition, could increase up to maximum 16 mg/day; doses divided 3-4 times/day. At hospital discharge, mean dose was 7.5 mg per day (range: 6–16 mg). The mean final dose for the	Main outcome was completion of the tapering dose or steady dose protocol at 6 months.<br><br>Open ended question to compare pain intensity compared to start of study; reported as “worse” or “better” (unvalidated scale).	No participants able to complete the taper; 5/6 able to complete 6 months of follow up in steady dose group (p=0.015).<br><br>Trial terminated early because of inability to taper.<br><br>2/12 lost to follow up.<br><br>8/10 (completed) were on long-term buprenorphine maintenance; 2/10	High ROB<br><br>(Cochrane tool)

© 2021 Powell VD et al. *JAMA Network Open.*

			11 participants remaining in the study was 9.8 mg per day (range: 4–16 mg).	Open ended question to describe overall level of function compared to start of study; reported as “worse” or “better” (unvalidated scale).<br><br>Only reported at 6 month follow up visit	returned to prior opioids.<br><br>8/10 who completed study reported improved pain; 8/10 who completed study reported improved daily life functioning.<br><br>Follow up: 6 months	
Nielsen et al. 2014.	POATS subgroup analysis of participants (n=569) who used extended -release oxycodone, methadone (≤40mg), immediate-release oxycodone, or hydrocodone.<br><br>Date: June 2006- July 2009	Case control design, secondary analysis of RCT<br><br>Evaluated whether longer-acting opioid formulations predicted increased withdrawal after first buprenorphine dose using t-tests, chi-square, and a linear regression model.	Participants on long acting opioids stopped 36 hours prior and those on short-acting stopped 12 hours prior to buprenorphine induction. Required COWS ≥8 before first dose of 4mg SL, followed by additional 4mg dose if withdrawal symptoms did not improve or worsen.<br><br>Discharged from clinic if COWS reduction of ≥2 points	COWS scores pre and 1 hour post induction, “difficult induction” defined as an increase in COWS after buprenorphine	Only 32/569 (6%) of inductions resulted in increased COWS. No significant difference across prescription opioid groups (p=0.47) or likelihood of a difficult induction (p=0.095); study was likely underpowered by low rate of difficult inductions.<br><br>Those with difficult inductions had lower pre-induction COWS scores (mean 10.1 vs 12.8, p<0.001).<br><br>A linear regression model only explained 7% of the variance, and was not able to predict difficult inductions.<br><br>Chronic pain showed a trend toward difficult	9/9<br><br>(NOS Case Control)

© 2021 Powell VD et al. *JAMA Network Open.*

					induction (56% of those with difficult inductions with chronic pain vs 41% of those without, p=0.085).<br><br>Follow up: N/A, examined induction period only.	
Worley et al, 2015.	POATS subgroup analysis restricted to those with chronic pain and participating in Phase 2 (n=125).<br><br>Date of study: June 2006-July 2009<br><br>.	Case Control<br><br>Subgroup analysis of a sequential adaptive RCT; evaluated effect of pain volatility and intensity during buprenorphine maintenance on likelihood of successful Phase 2 outcome (abstaining from opioids during the final week of buprenorphine maintenance and ≥2 of the 3 previous weeks) using hierarchical linear models.	From primary analysis of POATS:<br><br>Participants were instructed to stop short-acting opioid analgesics at least 12 hours prior to induction. Those previously on methadone (≤40mg) were instructed to stop 36 hours prior. When COWS score was ≥8, received 4 - 12 mg (in 4mg increments) on induction day.<br><br>At each subsequent visit, study physician could adjust the dose up to 8mg/week depending on withdrawal symptoms and adverse effects, but not for pain (range 8 - 32 mg/day.)<br><br>During the Phase 2 stabilization (12 weeks), the mean maximum dose was 20.3(SD=7.9, range 8-32mg/day) in a single daily dose.	Pain Intensity (BPI-SF), measured weekly.<br><br>For this study, restricted to the current pain severity item, which ranged from 0-10 (most severe).<br><br>Scores during maintenance phase were used to predict successful Phase 2 outcome (abstaining from opioids during the final week of buprenorphine maintenance and ≥2 of the 3 previous weeks).<br><br>Additional opioid use measured by self-report and urine toxicology.	Pain declined significantly over the 12 weeks of buprenorphine treatment (p<0.001)<br><br>Baseline pain severity was not associated with treatment outcome (p=0.52), but greater pain volatility predicted lower likelihood of successful phase 2 outcome (p<0.05)<br><br>A higher peak buprenorphine dose significantly associated with lower likelihood of success in Phase 2 (p<0.05).<br><br>Greater pain volatility associated with decreased odds of successful phase 2 outcome (OR=0.52, 95% CI 0.30, 0.89).<br><br>Follow up: 20 weeks total for Phase 2 (12	9/9<br><br>(NOS Case Control)

			Taper occurred in Phase 2 over 4 weeks (weeks 13-16.)		week buprenorphine maintenance, 4 week taper, 8 week follow up).	
Worley et al, 2017.	POATS subgroup analysis restricted to those with chronic pain and participating in Phase 2, with at least one follow up visit during a 4-week taper (n=125).<br><br>Date of study: June 2006-July 2009	Case Control<br><br>Subgroup analysis of an sequential adaptive RCT; evaluated effect of pain volatility on likelihood of subsequent additional opioid use while being tapered off buprenorphine using a multi-level growth curve model.	From primary analysis of POATS:<br><br>Participants were instructed to stop short-acting opioid analgesics at least 12 hours prior to induction. Those previously on methadone (≤40mg) were instructed to stop 36 hours prior. When COWS score was ≥8, received 4 - 12 mg (in 4mg increments) on induction day.<br><br>At each subsequent visit, study physician could adjust the dose up to 8mg/week depending on withdrawal symptoms and adverse effects, but not for pain (range 8 - 32 mg/day.)<br><br>During the Phase 2 stabilization (12 weeks), the mean maximum dose was 20.3(SD=7.9, range 8-32mg/day) in a single daily dose.<br><br>Taper occurred in Phase 2 over 4 weeks (weeks 13-16.)	Pain Intensity (BPI-SF), measured weekly. The Pain Intensity Scores during the buprenorphine maintenance phase were used to predict opioid use during the buprenorphine taper.<br><br>Additional opioid use measured by self-report and urine toxicology.	Mean pain intensity declined during the 12-week buprenorphine maintenance phase, but the decline was less in those who used opioids during the taper.<br><br>During the taper, additional opioid use increased from 22% at beginning to 31% and end of taper (p=0.001; (IRR=1.42, 95% CI 1.15,1.74)<br><br>Both increasing pain over time and volatility of pain predicted positive urine opioid results ; (OR 2.38 (p=0.02) and 2.43 (p=0.04), respectively)<br><br>Follow up: 20 weeks total for Phase 2 (12 week buprenorphine maintenance, 4 week taper, 8 week follow up).	9/9<br><br>(NOS Case Control)

© 2021 Powell VD et al. *JAMA Network Open.*

Weiss et al, 2014.	POATS subgroup analysis restricted to those participating in Phase 2 (n=360).<br><br>Date of study:<br>June 2006- July 2009	Case control<br><br>Subgroup analysis of a sequential adaptive RCT; evaluated whether adequate adherence to behavioral treatment adjusting for factors including chronic pain and adherence to buprenorphine treatment affected whether they were more likely to be successful at the end of the 12-week buprenorphine maintenance treatment using logistic regression models.	From primary analysis of POATS:<br><br>Participants were instructed to stop short-acting opioid analgesics at least 12 hours prior to induction. Those previously on methadone (≤40mg) were instructed to stop 36 hours prior. When COWS score was ≥8, received 4 - 12 mg (in 4mg increments) on induction day.<br><br>At each subsequent visit, study physician could adjust the dose up to 8mg/week depending on withdrawal symptoms and adverse effects, but not for pain (range 8 - 32 mg/day.)<br><br>During the Phase 2 stabilization (12 weeks), the mean maximum dose was 20.3(SD=7.9, range 8-32mg/day) in a single daily dose.<br><br>Taper occurred in Phase 2 over 4 weeks (weeks 13-16.)	Successful Phase 2 outcome defined as abstaining from opioids during the final week of buprenorphine maintenance and ≥2 of the 3 previous weeks.<br><br>Opioid dependence severity measured by Addiction Severity Index (ASI) composite score, presence of lifetime heroin use, and presence of current chronic pain.<br><br>Additional opioid use measured by weekly self-report and urine toxicology.<br><br>Adherence to behavioral treatment defined as attending ≥60% of offered counseling sessions.<br><br>Adherence to buprenorphine	N=177/360 (49.2%) had a successful outcome in Phase 2; success was not related to behavioral treatment condition (this result is a repeat from the primary POATS analysis).<br><br>Chronic pain was not associated with likelihood of successful Phase 2 outcome (OR 1.3, 95% CI 0.8-2.0, p=0.24).<br><br>Most participants (n=266 (74%) with adequate behavioral treatment adherence; chronic pain not associated (OR 0.5, 95% CI 0.2-1.5, p=0.5).<br><br>Adherence to buprenorphine treatment was high (95%).	8/9<br><br>(NOS Case-Control)

© 2021 Powell VD et al. *JAMA Network Open.*

				(total dose taken/total dose prescribed)		
Griffin et al, 2016.	POATS subgroup analysis restricted to those reporting chronic pain and participating in Phase 2 (n=148). Mean age 34.1 (range 18-61 years). 45% women. 55% employed full-time.<br><br>Date of study: June 2006-July 2009	Case Control<br><br>Subgroup analysis of an sequential adaptive RCT; evaluated effect of pain on likelihood of subsequent additional opioid use while taking buprenorphine.	From primary analysis of POATS:<br>Participants were instructed to stop short-acting opioid analgesics at least 12 hours prior to induction. Those previously on methadone (≤40mg) were instructed to stop 36 hours prior. When COWS score was ≥8, received 4 - 12 mg (in 4mg increments) on induction day.<br><br>At each subsequent visit, study physician could adjust the dose up to 8mg/week depending on withdrawal symptoms and adverse effects, but not for pain (range 8 - 32 mg/day.)<br><br>During the Phase 2 stabilization, the mean maximum dose was 20.3 (SD=7.9, range 8-32mg/day) in a single daily dose.	Pain intensity (BPI – SF), measured weekly. The Pain Intensity Score was used to predict opioid use in the subsequent week.<br><br>Additional opioid use (self-report and urine toxicology results)	During the 12 weeks of buprenorphine treatment, overall participants were other opioid-abstinent for 2/3 of weeks total.<br><br>Baseline pain severity mean 4.4 (SD=2.1), but it fluctuated for most participants during the study, with 68% crossing categories of mild, moderate, or severe pain, reporting no-mild pain at times and moderate-severe pain at other times.<br><br>Higher pain severity score in a given week significantly associated use of additional opioids in the subsequent week (adjusted OR 1.15 (95%CI 1.06-1.24,) p<0.001).<br><br>Follow up: 20 weeks total for Phase 2 (12 week buprenorphine maintenance, 4 week taper, 8 week follow up).	7/9<br><br>(NOS Case Control)

Baron et al, 2006.	Patients (n=23) referred for opioid detoxification because either they or treating physician felt were not getting benefit from current high dose opioid regimen. No one was referred for concerns for misuse or diversion. Mostly with musculoskeletal pain (83%). 9% with fibromyalgia and 9% with neuropathic pain. Dose range approximately 45 - >1,000 OME.<br><br>Date of study: March 2004 - May 2006	Cohort study.<br><br>Retrospective description of sequential patients who underwent inpatient opioid detoxification for decreased analgesic efficacy.	For those who received buprenorphine (n=16), received "loading" dose of 4mg every half-hour for the first three doses, followed by 4mg three times daily. Ibuprofen 200mg up to six times daily allowed.	Numeric Rating Scale (NRS) changes from prior to detoxification and post detoxification(after buprenorphine weaned).	Significant improvement in pain overall (p <0.001). 21/23 patients reported improved pain (mean pre-detox NRS 8 (SD 0.3), post-detox NRS 3.35 (SD 0.33))<br><br>Buprenorphine group with 63% reduction in pain compared to ibuprofen only group with 47% reduction (not significant.)<br><br>Pain scores were measured AFTER buprenorphine was weaned.<br><br>Follow up: non-systematic; up to 180 days.	6/9<br><br>(NOS Cohort)
Sturgeon et al, 2020.	Patients (n=240) with chronic pain on high-dose long term opioids who were referred to structured opioid refill clinic with mostly chronic low back pain (59.5%). Fibromyalgia was diagnosed in 7.9%, but 54% met ACR criteria. High psychiatric comorbidity with 78%, 64%, and 15% with depression, anxiety disorder, and PTSD, respectively. 27% concurrently prescribed benzodiazepine or	Cohort study<br><br>Description of referral clinic and outcomes along with risk factors. A taper was attempted to every patient; only moved to buprenorphine if unable to tolerate taper.	Buprenorphine transitions classified as "urgent" (evidence of aberrant behavior or urine toxicology results) or "nonurgent." For urgent transitions, individuals underwent withdrawal period of 24 hours (48 hours for fentanyl patches or methadone) and received tizanidine three times per day for withdrawal symptoms. For nonurgent transitions, patients were provided with a schedule and	Change in pain after taper or after initiating buprenorphine via NRS; completion rates (either of taper or initiation of buprenorphine).	N=107 successfully tapered; pain intensity increased significantly (p=0.005).<br><br>N=45 successfully transitioned to buprenorphine; median initial pain intensity 5.93, median follow up pain intensity 5.58, not significant, p=0.66.<br><br>Those who transitioned to buprenorphine had higher baseline OME doses than those who	6/9<br><br>(NOS Cohort)

	sedative. Opioid dose median 182 OME (range 100 - 3,200 OME) daily. Mean age 57.8 year (range 25 – 93). 57% women. N=144 disabled (60%).<br><br>Date of study: Data collection started in 2016		provided gabapentin ≤900mg/day, tizanidine 2mg three times per day on the day of withdrawal, then scheduled to transition on the day after they ran out of prior opioid. They were counseled regarding the process, including the risk of precipitated withdrawal. For those with high anxiety related to withdrawal, quetiapine 50mg three times daily was initiated during the 24-48 hour withdrawal period.		tapered successfully (median 265 vs 173 OME, p<0.001).<br><br>49% who transitioned to buprenorphine had improvement in pain.<br><br>N=88 dropped out; these had a higher initial dose compared with taper (p=0.033) and used more benzodiazepines (p=0.018).	
Aurilio et al., 2009.	n=32 patients with chronic cancer pain, treated with either buprenorphine patch (70µg/hr, n=16) or fentanyl patch (75µg/hr, n=16) for the prior three months with inadequate pain relief, and adverse events related to opioid treatment, including sedation, dysphoria, nausea/vomiting and constipation. Mean age 62 (range 42-78) years. N=15 women.	Uncontrolled pre-post<br><br>Patients using fentanyl patch 75µg/hr were rotated to 52.5µg/hr buprenorphine patch; those using buprenorphine patch (70µg/hr) rotated to a 25µg/hr fentanyl patch.	Removed old patch (either fentanyl or buprenorphine) and immediately applied patch containing the new medication (either buprenorphine or fentanyl) to a different site. Morphine 20mg tablets as a "rescue" medication up to three times/day.	Primary outcome; pain reduction via Visual Analog Scale (VAS, measured as weekly mean of daily scores), Present Pain Intensity (PPI, measured at weekly visits). Pain Rating Index (PRI) from Short-Form McGill Pain Questionnaire containing sensorial and emotional sphere of pain.<br><br>Secondary outcome was reduction of	VAS and PPI scores reduced significantly (p <0.0001) in both groups. In buprenorphine group, VAS scores decreased by 69%; PPI scores reduced by 79% by the end of study (p<0.0001).<br><br>PRI scores reduced significantly (p <0.0001) in both groups.<br><br>In buprenorphine group, PRI scores decreased by 62% (p<0.0001)	Inherently high ROB due to study design (uncontrolled pre-post)

© 2021 Powell VD et al. *JAMA Network Open.*

				rescue morphine in milligrams.<br><br>Investigator assessment of nausea/vomiting, constipation, dysphoria, and 4 point unvalidated sedation scale (0 = no sedation, 3= severe sedation)	Significant (p<0.0001) reduction in rescue morphine use.<br><br>In buprenorphine group, number of patients with nausea/vomiting reduced from n=8 to n=2; constipation n=11 to n=4; dysphoria n=2 to n=0.<br>Those with no sedation improved from n=12 to n=16 (all).<br><br>Follow up: 4 weeks.	
Berland et al, 2013.	n=76 with chronic pain who experienced worsening pain and function despite escalating opioid doses (median 400 OME daily). Most common pain types were musculoskeletal back pain (33%) and widespread/fibromyalgia (30%). Median age 48 (range 19-82) years. N=44 women.<br><br>Date of study: 2009 - 2010	Uncontrolled pre-post<br><br>Description of one group's experience rotating patients with chronic pain and opioid dependence to buprenorphine.<br><br>All participants received buprenorphine.	Induction with IM or SL, then SL conversion (naloxone presence not specified). Dosed 2-4 times/day. Daily SL buprenorphine dose at discharge was 2-20 mg (median 8mg).	Non-validated three category assessment of pain intensity change: "much better, good, pain free;" "fair, better, tolerable;" or "no improvement, not great, worse."<br><br>Functional state changes ranged from "much better" to "no change, worse or no report"; unvalidated scale<br><br>Clinical judgement/ assessment of withdrawal	2/3 reported improved pain. "much better, good, pain free" n=26 (34%); "fair, better, tolerable" n= 25 (33%); "no improvement, not great, worse" n=25 (33%)<br><br>"Much better" or "somewhat better" functional state in n=46 (60%)<br><br>New employment in n=5 (7%)<br><br>No individuals with provoked opioid withdrawal, toxicity, deaths from conversion; 2 unrelated deaths	Inherently high ROB due to study design (uncontrolled pre-post)

© 2021 Powell VD et al. *JAMA Network Open.*

				Number of patients remaining on buprenorphine or returning to other opioids	Remained on buprenorphine: n=41 (54%)<br><br>Returned to full agonist opioid: n=16 (21%)<br><br>Follow up: non-systematic, 0-25 months (median 8 months)	
Daitch et al, 2012.	Patients (n=104) with chronic pain who experienced continued or worsening pain despite use of opioids who were seen at an interventional pain management practice. Most commonly used opioid was oxycodone (45%). Dose mean 180 (range 10 – 840) OME. Mean age 49 (range 21-78 years). 42% women.<br><br>Date of study: December 2007 - July 2010	Uncontrolled pre-post<br><br>Description of one pain clinic's experience rotating patients to buprenorphine who remained on buprenorphine for ≥60 days.<br><br>All participants received buprenorphine.	Stopped prior opioids at least 24 hours prior to buprenorphine (48 - 72 hours for those switching from methadone). Initial dose of 8mg SL. Patients instructed to take additional 8mg dose after one hour if pain/withdrawal symptoms continued, not to exceed 32mg daily. The dose was titrated based on patient report after one week.	NRS (primary outcome) before and 2 months after buprenorphine<br><br>Adverse effects reporting (nonsystematic)	Significant reduction in pain after 2 months of buprenorphine treatment (mean 2.3 points, p<0.001).<br><br>Mean 2.3 point reduction via NRS overall, P<0.001<br><br>Those who used >400 OME prior to rotation had lower decrease (mean 1.1 point decrease). Those who used 100-199 OME per day had higher decrease (mean 2.7 point decrease in pain).<br><br>Most common side effects were nausea, dizziness, urinary retention, and sexual dysfunction. No frequencies given.	Inherently high ROB due to study design (uncontrolled pre-post)

© 2021 Powell VD et al. *JAMA Network Open.*

					Follow up; non-systematic, remained on buprenorphine for mean 10.3 months (range 2-42 months)	
Daitch et al, 2014.	Patients (n=35) with chronic pain who experienced continued or worsening pain despite use of high-dose opioids who were seen at an interventional pain management practice. Age range 24 – 66 (mean 49) years. 40% women. Dose mean 550 (range 200 - 1,370).<br><br>Date of study: July 2010 - April 2011	Uncontrolled pre-post<br><br>Description of one pain clinic's experience rotating patients from high dose opioids to SL buprenorphine who remained on buprenorphine for ≥60 days.<br><br>All participants received buprenorphine (mean dose 28.11 mg /day, SD = 5.94.)	Prior opioids stopped ≥ 24 hours (48-72 hours for transdermal fentanyl and methadone). After participants achieved a COWS ≥13, they were given an initial dose of 8mg. Patients instructed to take additional 8mg dose after one hour if pain/withdrawal symptoms continued, not to exceed 32mg daily. The dose was titrated based on patient report after one week. Used clonidine during first week.	Primary outcome was reduction in pain via NRS After 2 months on buprenorphine<br><br>Pre-induction COWS (although scores not reported)	Significant decrease in pain (7.2 to 3.5 points; p<0.001), with 34/35 patients reporting decreased pain.<br><br>Across dose range, there was a mean 61% reduction in pain.<br><br>All patients experienced withdrawal symptoms the first week and received clonidine; no information regarding COWS scores and/or changes<br><br>Follow up: mean treatment duration 6 months (non-systematic follow-up)	Inherently high ROB due to study design (uncontrolled pre-post)
Freye et al, 2006.	Patients (n=42) with inadequate pain relief or severe side effects on long-term opioids. Pain primarily musculoskeletal (64%); chronic cancer pain (21%); neuropathic pain (19%). Dose range 120 - 800 OME/day. Age range 34 – 86 years. N=19 (45%) women.	Uncontrolled pre-post<br><br>Open label prospective<br><br>All participants received buprenorphine patch.	Varied starting dose by groups based on prior opioid use (Group 1: 120 mg morphine<br>Group 2: 121-240 mg<br>Group 3: >240 (mean >400mg/day)) range of starting dose range of "<52.5µg/hr" (n=1) to 105µg/hr (n=1). Most (69%) started with 52.5µg/hr. 71% remained	5 point unvalidated scale of pain relief (0 = very good; 4 = no pain relief).<br><br>5 point unvalidated scale of sleep quality (0=very good; 4 = very poor).	Pain improved significantly (p<0.005).<br><br>Percent of patients reporting "good" or "very good" pain relief increased from 5% prior to rotating to buprenorphine to 76%; "poor" to "satisfactory"	Inherently high ROB due to study design (uncontrolled pre-post)

© 2021 Powell VD et al. *JAMA Network Open.*

			on initial dose, but n=10(24%) required an increased dose (maximum 140µg/hr.)	Reported type and severity of side effects as well as reasons for termination of therapy	pain relief in 95% to 17%.<br><br>Improved quality of sleep was found in 74% ($p>0.005$).<br><br>Significant reduction in number of patients requiring laxatives ($p<0.001$). Main side effects were local skin reactions due to patch (12%) and one patient had hyperhidrosis (2.4%). No other serious AEs observed; none resulted in study withdrawal.<br><br>Follow up: nonsystematic, ranged from 10 weeks to one year.	
Malinoff et al, 2005.	Patients (n=95) with worsening pain and function despite opioid dose escalation. Opioids used mean 8.8 years (range 1.5 - 27 years). Mean age 51.3 (range 26-84) years. 48% women. 71% employed.<br><br>Date of study: December 2003 - October 2004	Uncontrolled pre-post<br><br>Description of a pain clinic's experience rotating patients to SL buprenorphine.<br><br>All participants received SL buprenorphine	Participants stopped prior opioid ≥12 hours pre-induction. Individuals given a "test" dose of 1mg buprenorphine/0.25 naloxone and observed for withdrawal; then administered two doses of 2mg/0.5mg at 45 minute intervals. Discharged about 2 hours after starting treatment. They were seen in clinic 3-5 days after initial start, followed by phone, and seen at	5-level VAS before and after treatment.<br><br>Rates of side effects and discontinuation of treatment, along with reason.	86% of patients with substantial pain improvement.<br><br>VAS mean pre-treatment 3.9 (SD=0.4); mean post treatment 2.2 (SD=0.5)<br><br>Ataxia/lightheadedness n=12 (13%); nausea n=9 (10%); cephalgia n=15 (16%).<br><br>n=6 patients (6.25%) discontinued	Inherently high ROB due to study design (uncontrolled pre-post)

© 2021 Powell VD et al. *JAMA Network Open.*

			least monthly. Final dose mean 8mg (range 2-20mg buprenorphine/naloxone).		buprenorphine due to side effects<br><br>Follow up: nonsystematic, mean 8 months (range 2.4 – 16.6 months)	
Pade et al, 2012.	Veterans (n=143) with chronic pain and high risk opioid use (including high-dose or complex therapeutic pain regimens) who were referred to a special clinic. Pain mostly musculoskeletal, with 39% having mixed nociceptive/neuropathic, and 4% with fibromyalgia. Median duration of prior opioid use 105 months (range 3-240 months) Most common opioids were oxycodone (44%), methadone (16%). 16 (11%) were current heroin users. Median daily dose 120 OME (range 30-375 OME). 93% men; mean age 52 years (range 26-75).<br><br>Date of study: July 2009 - December 2011	Uncontrolled pre-post<br><br>Data collected as part of a quality improvement initiative for a single clinic.<br><br>All participants received SL buprenorphine (mean total daily dose 16 mg, range 6-28 mg).	Individuals on long-acting opioids were tapered to <90 OME, then switched to a short acting opioid from 2-4 weeks prior to induction. Used a previously published protocol for buprenorphine rotation.	Mean NRS pain scores pre and post (mean of 5 pre and post induction scores).<br><br>Retention in treatment / percent who continued	Mean pain scores decreased from 6.39 to 5.6 (p<0.001).<br><br>65% who started on Bup/nx continued treatment and had not restarted opioids. 65% of those patients stayed on bup/nx for >6 months, and 6% >18 months. 16% of those who discontinued did so due to ongoing pain complaints.<br><br>Follow up: Unspecified, in some cases >18 months.	Inherently high ROB due to study design (uncontrolled pre-post)
Rosenblum et al. 2012.	n=12 with chronic pain who demonstrated ≥one aberrant behavior but no current diagnosable substance use disorder. Most patients had pain lasting >5 years with	Uncontrolled pre-post<br><br>Evaluation of a pilot protocol for rotation to buprenorphine.	Instructed to discontinue opioids the night before induction (those taking ≥30 mg methadone instructed to stop two days before.) Induction dose 2mg SL with	Pain intensity/severity via BPI subscale (0-10). Pain interference with function via BPI subscale (0-10).	Average and worst pain significantly declined from baseline (Average pain mean 6.6 to mean 2.1, p<0.01; Worst pain declined from mean 8.2 to mean 4.8, p<0.01)	Inherently high ROB due to study design (uncontrolled pre-post)

© 2021 Powell VD et al. *JAMA Network Open.*

	diverse etiologies, including degenerative disc disease, neuropathic, and complex regional pain syndrome (CRPS). Duration of opioid use: 1-25 years (mean 8.5, SD 7.3). Dose range 15-450 OME. Mean age 50 years (range 40-66 years); 42% women.<br><br>Date of study: July 2008 - February 2010	All participants received buprenorphine (final mean dose 12mg (range 8 - 24 mg). Dosed three times/day.	naloxone, allowing subsequent doses up to 20mg.	COWS and SOWS, measured before and after buprenorphine.<br><br>Percent who completed study protocol .<br><br>Adverse event reporting.	Pain interference with function (available in n=4) declined in 2 but increased mildly in 2; improvement was seen on average (mean pre 6.00 to post 2.86.)<br><br>Pre-buprenorphine COWS mean 11.25 (SD 7.24) and SOWS mean 21.5, (SD 14.64); post first dose COWS mean (7.58, SD 5.99). SOWS mean 14.83, SD 16.63).<br><br>However, four patients with severe withdrawal, one hospitalized and others visited ED. Three of these on >300 OME/day.<br><br>Only n=4 completed study; n=8 withdrew. N=7 stopped secondary to adverse events/side effects; n=1 stopped because no longer had pain.<br><br>10/12 reported adverse events, requiring 7 to stop treatment as a result. These included sedation, dizziness, confusion, thirst/dry	

					mouth, slurred speech, among others.<br><br>Early study cessation due to safety concerns.<br><br>Follow up: 3 – 6 months	
Streltzer et al, 2015.	Patients (n=43) with chronic, difficult-to-control pain referred to pain clinic if there was suspicion of psychological factors affecting pain, or primary care physician (PCP) was uncomfortable with opioid management. Patients were preoccupied with maintaining prescriptions and hesitant to reduce dose; not necessarily taking opioids in excess of prescribed dose. Dose range 30 - 1,440 OME, most (93%) taking >120 OME. 47% also were co-prescribed benzodiazepines. 49% had a history of other substance abuse. Age range 45-60. 30% Women; N=6 (14%) employed.<br><br>Date of study: January 1, 2006 - December 30, 2008	Uncontrolled pre-post<br><br>Retrospective chart review of patients who received SL buprenorphine at a single pain clinic.<br><br>Final buprenorphine dose median 8mg (range 0.25 – 32 mg).	The starting dose of buprenorphine ranged from 2 - 32mg (median 12mg), with the majority (56%) starting with 8-16mg.	Maintenance of buprenorphine	Median treatment duration 19 months (range 1 – 85 months)<br><br>N=35 and n=26 maintained treatment ≥6 and ≥12 months, respectively.<br><br>One patient died of overdose after discontinuing buprenorphine.<br><br>10/43 (23%) returned to using prescription opioids, drop out, or were transferred to licensed opioid treatment program.<br><br>Follow up: 1 – 85 months, nonsystematic	Inherently high ROB due to study design (uncontrolled pre-post)
Tang et al., 2020.	Patients with OUD or opioid dependence due to chronic pain (n=23 total, n=9 with chronic pain) who were transitioned to buprenorphine during an inpatient stay. Chronic pain	Uncontrolled pre-post<br><br>Retrospective chart review of one hospital's experience rotating inpatients to buprenorphine, some of	Participants received a "microdose" transdermal (5-20mcg/hr) as a bridge to higher doses in an effort to avoid withdrawal After 12 - 48 hours, administered 2-4mg SL,	COWS (available for n=9); chart documentation of withdrawal<br><br>Retention in hospital stay,	n=5 with documentation of mild and tolerable withdrawal symptoms via chart notes. n=15 with no symptoms.	Inherently high ROB due to study design (uncontrolled pre-post

	group used opioids for a mean 7.2 years, mean daily OME 379.<br><br>Date of study: January 2015 - December 2016	whom had chronic pain-related opioid dependence.<br><br>All participants received buprenorphine.	followed by rapid titration of 2-4mg until withdrawal symptoms resolved. Process occurred over 2-6 days.	number discharged on SL buprenorphine, number on SL buprenorphine in 4 weeks	Mean COWS prior to patch application 7.0 (SD 4.8, n=4); mean COWS 24 hours after patch 8.9 (SD 3.7, n=6), Mean COWS 24-48 hrs post 5.1 (SD 5.0, n=5).<br><br>N=22 did not leave AMA; n=17 discharged on SL buprenorphine (mean dose 12mg); n=10 using SL buprenorphine at 4 weeks (including n=5/9 with chronic pain). One patient died of unknown cause.<br><br>Follow up: 4 weeks	

eTable 2. Details of GRADE Scores by Outcome

Outcome	*n* studies	Initial GRADE score	Adjustment	Reason for Adjustment	Final GRADE Score
**Precipitated Opioid Withdrawal**	7				
RCT	2	4	-2	-2 Risk of bias (very serious)<br>-1 Indirectness (serious)<br>+1 Dose response	2 Low
Controlled observational[1]	1	2	0		2 Low
Other	4	1	0	-2 Risk of bias (very serious)<br>-2 Indirectness (very serious)	1 Very Low
**Pain Intensity/Severity**	17				
RCT	4	4	-2	-2 Risk of bias (very serious)<br>-1 Indirectness (serious)<br>+1 Dose response	2 Low
Controlled observational	5	2	-1	-1 Risk of bias (serious)<br>-2 Indirectness (very serious)	1 Very Low
Other	8	1	0	-2 Risk of bias (very serious)<br>-1 Indirectness (serious)<br>+1 Large effect	1 Very Low
**Pain Interference**	4				
RCT	2	4	-3	-2 Risk of bias (very serious)<br>-2 Indirectness (very serious)	1 Very Low
Other	2	1	0	-2 Risk of bias (very serious)<br>-2 Indirectness (very serious)	1 Very Low
**Completion of Protocol/Continuation of Treatment**	14				
RCT	5	4	-3	-2 Risk of bias (very serious)<br>-1 Indirectness (serious)	1 Very Low
Controlled observational	3	2	-1	-1 Risk of bias (serious)<br>-1 Indirectness (serious)	1 Very Low
Other	6	1	0	-2 Risk of bias (very serious)<br>-2 Indirectness (serious)	1 Very Low
**Side Effects/Adverse Events**	9				
RCT	4	4	-3	-2 Risk of bias (very serious)<br>-1 Indirectness (serious)	1 Very Low
Other	5	1	0	-2 Risk of bias (very serious)<br>-1 Indirectness (serious)	1 Very Low
**Mental Health**	2				

© 2021 Powell VD et al. *JAMA Network Open.*

RCT	1	4	-3	-2 Risk of bias (very serious)<br>-1 Indirectness (serious)	1 Very Low
Other	1	1	0	-2 Risk of bias (very serious)	1 Very Low
**Health Care Utilization**	0	No data available			

© 2021 Powell VD et al. *JAMA Network Open.*

eTable 3. Cochrane Risk of Bias Assessment Tool Results for Randomized Controlled Trials (RCTs)

	**Random sequence generation (selection bias)**	**Allocation Concealment (selection bias)**	**Blinding of participants and personnel (performance bias)**	**Blinding of outcome assessment (detection bias)**	**Incomplete Outcome data (attrition bias)**	**Selective Reporting (reporting bias)**	**Overall Risk of Bias**
Blondell et al, 2010	Low	High	High	High	High	Some concerns	High
Neumann et al, 2019	Some concerns	High	High	High	High	Some concerns	High
Roux et al, 2013	Low	Low	Low	Low	High	Some concerns	High
Webster et al, 2016	Some concerns	Low	Low	Some concerns	High	Some concerns	High
Weiss et al., 2011	Low	Low	Some concerns	Low	Low	Low	Some concerns

© 2021 Powell VD et al. *JAMA Network Open.*

eTable 4. Newcastle Ottawa Scale Risk of Bias Assessment for Case Control Studies

	Selection (max 4 stars)				Comparability (max 2 stars)	Outcome (max 3 stars)			
	**Adequate Case Definition**	**Case Representativeness**	**Selection of Controls**	**Definition of Controls**	**Comparability**	**Ascertainment of Exposure**	**Same Method of Ascertainment for Cases and Controls**	**Non-Response Rate**	**Total**
Griffin et al, 2016	Yes, with independent validation *	Potential for selection bias	Community controls *	No history of the outcome *	Study controls for pain, opioid use, and substance use disorder variables AND other variables **	Structured patient interview where interviewer is blind to case/control status *	Yes *	Non-response rate different for cases and controls and no designation	7
Nielsen et al, 2014	Yes, with independent validation *	Consecutive or obviously representative series of cases *	Community controls	No history of the outcome *	Study controls for pain, opioid use, and substance use disorder variables AND other variables **	Secure record *	Yes *	Same for both groups *	9
Weiss 2014	Yes, with independent validation *	Consecutive or obviously representative series of cases *	Community controls *	No history of the outcome *	Study controls for pain, opioid use, and substance use disorder variables AND other variables **	Secure record *	Yes *	Non-response rate different for cases and controls and no designation	8
Worley 2015	Yes, with independent validation *	Consecutive or obviously representative series of cases *	Community controls *	No history of the outcome *	Study controls for pain, opioid use, and substance use disorder variables AND other variables **	Secure record *	Yes *	Same for both groups *	9
Worley 2017	Yes, with independent validation *	Consecutive or obviously representative series of cases *	Community controls *	No history of the outcome *	Study controls for pain, opioid use, and substance use disorder	Secure record *	Yes *	Same for both groups *	9

© 2021 Powell VD et al. *JAMA Network Open.*

					variables AND other variables **				

	Selection (max 4 stars)	Comparability (max 2 stars)	Outcome (max 3 stars)
Griffin et al, 2016	☼☼☼	☼☼	☼☼
Nielsen et al, 2014	☼☼☼☼	☼☼	☼☼☼
Weiss et al, 2014	☼☼☼☼	☼☼	☼☼
Worley et al, 2015	☼☼☼☼	☼☼	☼☼☼
Worley et al, 2017	☼☼☼☼	☼☼	☼☼☼

© 2021 Powell VD et al. *JAMA Network Open.*

eTable 5. Newcastle Ottawa Scale Risk of Bias Assessment for Cohort Studies

	Selection (max 4 stars)				Comparability (max 2 stars)	Outcome (max 3 stars)			
	**Representativeness of Exposed Cohort**	**Selection of the Non-Exposed Cohort**	**Ascertainment of Exposure**	**Outcome Not Present at Start of Study/ Baseline**	**Comparability**	**Assessment of Outcome**	**Follow-up Length**	**Follow-up Adequacy**	**Total Quality Score**
Baron et al, 2006	Selective group	Drawn from Same Community as Exposed Cohort *	Secure Record *	Yes *	No	Record linkage *	Yes *	Complete follow – up *	6
Sturgeon et al, 2020	Somewhat representative *	Drawn from a Different Source	Secure Record *	Yes *	No	Record linkage *	Yes *	Subjects lost to follow-up unlikely to introduce bias *	6

	Selection (max 4 stars)	Comparability (max 2 stars)	Outcome (max 3 stars)
Baron et al, 2006	☼☼☼		☼☼☼
Sturgeon et al, 2020	☼☼☼		☼☼☼

© 2021 Powell VD et al. *JAMA Network Open.*

eTable 6. Details of Buprenorphine Rotation Protocols[1]

Study	Setting of rotation	Indication for rotation, including presence of OUD and whether rotation was voluntary or required as condition of care	Baseline opioid (dose range)	Abstinence period prior to induction?	Formulation (s)	Starting dose and frequency	Additional medication provided during transition?	Maximum and/or final dose	Intent to continue or taper
Aurilio et al., 2009.	Outpatient	Inadequate analgesia and side effects of current medication (sedating, dysphoria, nausea/vomiting, constipation) Use of any psychoactive substance was an exclusion criterion. Transition was voluntary.	Transdermal fentanyl (75µg/hr)	None; fentanyl patch was removed and buprenorphine patch placed on a different site simultaneously	Transdermal	52.5µg/hr continuous application; no titration<br><br>Chosen to represent estimated 50% equianalgesic dose	oral morphine (immediate release) 20mg, up to three times daily allowed for the duration of the study	52.5µg/hr continuous application	Continued
Baron et al, 2006.	Mixed; 16/23 patients required inpatient admission	Patient and/or referring physician perceived inadequate benefit from current opioid regimen. No concern for misuse or OUD.	Oxycodone extended release (240-1200 mg/day); methadone (60-260mg/day); morphine (120-580mg/day);	Not reported	Sublingual	4mg every half hour for first three doses, followed by 4mg three times daily	Ibuprofen 200mg up to six times/day during detoxification	n/a	Tapered over ≤180 days

© 2021 Powell VD et al. *JAMA Network Open.*

		Voluntary transition.	transdermal fentanyl (50-400 μg/hr); meperidine (150mg/day); hydrocodone (45-150 mg/day); Approximately 45 - >1000 oral morphine equivalents (OME).						
Berland et al, 2013.	Inpatient	Inadequate analgesia and function with escalating opioid dose Evidence of OUD in n=18 (24%).<br><br>Voluntary transition.	Median 400 OME/day	Discontinued transdermal fentanyl or methadone 5 days prior to induction and replaced with morphine (200mg/day) or hydromorphone (40mg/day); other patients stayed on prior opioid until admission. For other patients, patient-controlled analgesia (PCA) system was discontinued for ≥6 hours until signs and/or symptoms of withdrawal appeared.	Induction with IM or sublingual, then maintained on sublingual	IM: 0.3mg q6 hours until withdrawal symptoms improved (1-4 doses); then transitioned to 6-12 mg sublingual in 3-4 divided doses daily.<br><br>Sublingual: 1mg q30 minutes for 4 doses, then 2mg q4 hours for 3-4 doses.	For those who used fentanyl patches or methadone, rotation to a morphine or hydromorphone PCA was used during the clearance period. Phenobarbital was substituted for benzodiazepines and other sedatives.	Dose at discharge range 2-20mg (median 8mg)	Continued
Blondell et al, 2010	Inpatient	Participants all had a self-described	Hydrocodone, oxycodone, methadone,	Midnight before hospital admission	Sublingual combination	After staff noted signs of withdrawal, 4mg administered,	Permitted to use non-opioids for	Mean final dose 9.8 mg/day	Randomized to half with taper

		chronic pain-induced addiction to prescription opioids and were seeking treatment. Participants were randomized to taper or steady-dose. Taper group was allowed to opt out of taper (and continue steady dose) at any time during the first 4 months.	morphine, fentanyl; dose range not reported		with naloxone	followed by 2mg q2 hours until withdrawal controlled.<br><br>Total amount required was given in 3-4 divided doses the next day (usually 2/0.5mg 3-4 times/day)	breakthrough pain	(range 4 – 16 mg/day); 2 – 4 divided doses	and half continued
Daitch et al, 2012.	Outpatient	Inadequate or worsening pain with opioid therapy. Participants consented to transition to buprenorphine. No information regarding presence/absence of OUD.	Oxycodone, fentanyl, hydrocodone, methadone, oxymorphone, morphine; Mean 180 OME/day (range 10-840 OME)	≥24 hours (48-72 hours for methadone)	Sublingual combination with naloxone	8mg initially followed by another 8mg one hour later if pain or withdrawal symptoms did not abate, not to exceed 32mg/day.	Not reported	Maximum allowed 32mg/daily	Continued
Daitch et al, 2014.	Outpatient	Continuous or worsening pain despite high-dose opioid therapy. Participants consented to transition to buprenorphine.	Mean 550 OME/day (range 200 - 1,370). Oxycodone, hydromorphone, oxymorphone, fentanyl,	≥ 24 hours (48-72 hours for transdermal fentanyl and methadone).	Sublingual	After participants achieved a COWS ≥13, they were given an initial dose of 8mg. Patients instructed to take additional 8mg dose after one hour if pain/withdrawal	Oral clonidine during first week	Final mean dose 28.11 (SD 5.94) mg/day; maximum allowed 32mg/daily	Continued

		No information regarding presence/absence of OUD.	methadone, morphine.			symptoms continued, not to exceed 32mg daily.			
Freye et al, 2006	Mixed	Inadequate analgesia or intolerable side effects. No information regarding voluntary or contingent nature of transition available. No mention of OUD, but “insufficient compliance” with prior opioid therapy was noted in 19%.	Range 120 – 800 mg OME/day	Not described	Transdermal	Range <52.5µg/hr - 105µg/hr.	Not described	Dose range <52.5 - 140µg/hr	Continued
Malinoff et al, 2005.	Outpatient	Worsening pain and function despite increasing opioids. Approximately 8% with OUD. Transition was voluntary.	Dose range not specified other than “high”	≥12 hours	Sublingual combination with naloxone	Test dose of 1/0.25mg buprenorphine/naloxone given and observed for signs of withdrawal; followed by 2 doses of 2/0.5mg q45 minutes.	Not described	Dose range 2-20mg (mean 8mg) in divided doses	Continued
Neumann et al, 2020.	Outpatient	Participants all had a self-described chronic pain-induced addiction to prescription opioids and were seeking	Dose range not specified	Midnight before starting buprenorphine	Sublingual combination with naloxone	Per a preliminary report, given 4mg dose followed by additional 2mg 2-4 hours later if withdrawal symptoms continued	Permitted to use non-opioids for breakthrough pain	8-16mg in 2-4 divided doses.	Continued

© 2021 Powell VD et al. *JAMA Network Open.*

		treatment. . Participants consented to rotation to either buprenorphine or methadone (randomized), and were allowed to switch to the other study drug on request.							
Pade et al, 2012	Outpatient	Participants were referred to a specialized clinic for those with co-occurring chronic pain and substance use disorder, where they were monitored closely. If there was evidence of aberrant medication use, they were recommended to rotate to buprenorphine; no information regarding whether this was voluntary or contingent on receiving care. Buprenorphine	Median daily dose 120 OME (range 30-375 OME). Most common opioids were oxycodone (44%), methadone (16%). 16 (11%) were current heroin users.	12-24 hours for short-acting opioids; 24-36 hours for long-acting opioids<br><br>For long-acting opioids, patients tapered to <90 OME and switched to equivalent short-acting opioid for 2-4 weeks	Sublingual combination with naloxone	Used Treatment Improvement Protocol (TIP) Series 40 protocol (Center for Substance Abuse Treatment, 2004)<br><br>Maintenance dosed 3-4 times/day	Not described	Mean dose 16mg (SD 5.4), range 6-28mg; administered in divided doses.	Continued

© 2021 Powell VD et al. *JAMA Network Open.*

		was continued unless patient returned to opioid use, had aberrant urine drug screens, or missed multiple visits or made multiple early refill requests, at which point they were referred for more intense OUD treatment.							
Rosenblum et al, 2012.	Outpatient	Participants all had aberrant behavior(s) regarding prescribed opioids for chronic pain, but meeting criteria for OUD (or other SUD) was an exclusion criteria. . Participants consented for transition.	Dose range 15-450 OME.	Instructed to discontinue 2:00am the day of induction; those taking methadone ≥30mg/day instructed to stop ≥2 days before	Sublingual combination with naloxone	When mild withdrawal symptoms developed, 2mg was administered, followed by 2-4mg at 1-2 hour intervals for 2-3 subsequent doses	Extra tablets (2-4mg) could be used for breakthrough pain, up to 4 times/day.	Mean 12 mg (range 8 – 24mg)/day ; divided into 3 doses.	Continued
Roux et al, 2013.	Inpatient	Participants all had co-occurring chronic pain and opioid dependence by DSM-IV criteria, but were not seeking	Primarily oxycodone, hydrocodone, hydromorphone, and tramadol. Dose median 60 OME (IQR 38-144)	Not described	Sublingual combination with naloxone	Randomized to 2, 8, or 16mg/day in 4 divided doses. No details regarding transition described.	Supplemental medications for emergent withdrawal symptoms (no further description provided)	All subjects received 2, 8, and 16mg in random order. Received in 4	Only provided for duration of study

		treatment for OUD. Buprenorphine was continued for only two weeks for each dose condition, which participants all received in random order. Participants gave consent for the study, and the intent was not long-term treatment.						divided doses.	
Streltzer et al, 2015.	Outpatient	Chronic pain complicated by psychological factors; difficult medication management (polypharmacy, etc) All participants met DSM-IV criteria for opioid dependence, however, authors noted that subjects often took opioids exactly as prescribed. Switching to buprenorphine was voluntary. After stabilization, patients were	Dose range 30-1,440 OME/day	Not described	Sublingual combination with naloxone	Starting dose ranged from 2 - 32mg (median 12mg), with the majority (56%) starting with 8-16mg.	Not described	Dose range 0.25 – 32 mg (median 8mg)	Continued

		allowed to reduce or taper buprenorphine dose at their own pace, or maintain steady dose.							
Sturgeon et al, 2020.	Outpatient	Patients Using high-dose, long-term opioids for chronic pain gave consent to be referred to a specialized clinic, where they were informed that they would undergo opioid taper or rotate to buprenorphine at the discretion of the prescriber. A taper to ≤90 OME was attempted; if unsuccessful, buprenorphine rotation was initiated. If patients refused, they returned to prior provider.	Dose median 265 OME/day (IQR 180-375)	Urgent transition: 24 hours; 48 hours for transdermal fentanyl and methadone<br><br>Nonurgent: one day after remaining opioids ran out.	Sublingual	Not described	Urgent transition: tizanidine 2mg TID<br><br>Non-urgent transition: gabapentin up to 900mg/day for the month prior; tizanidine 2mg TID on the day of withdrawal; quetiapine 50mg TID for those with anxiety related to the transition (1-2 days)	Median dose 4mg/day (range 3 – 8 mg/day)	Continued
Tang et al., 2020.	Inpatient	Patients had co-occurring chronic pain and opioid	Dose mean 379.4 OME/day	Short-acting opioids: approximately 12 hours.	Transdermal as bridge to higher-dose sublingual	5-20µg/hr patch applied for ≥12 hours; then started sublingual	Not reported	Mean dose 11.7mg/day	Continued

© 2021 Powell VD et al. *JAMA Network Open.*

		dependence. No information was available regarding consent to buprenorphine rotation and whether ongoing treatment was contingent on continuing.		Those previously on methadone rotated to morphine immediate-release prior to patch application.		buprenorphine 2-4mg, followed by 2-4mg additional doses until withdrawal symptoms resolved (2-6 days)			
Weiss et al, 2011. (and POATS secondary analyses)	Outpatient	All participants had prescription opioid dependence and consented to the study; they were stratified by chronic pain presence. If participants failed Phase I (brief 2 week taper), they were invited to phase 2 (12 weeks of maintenance /steady-dose phase followed by 4 weeks of taper and 8 weeks follow up). The taper was not voluntary.	Not described, though methadone was ≤40mg.	Short-acting opioids: 12 hours<br><br>Longer acting opioids (methadone≤40 mg): 36 hours	Sublingual combination with naloxone	When COWS ≥8, 4mg administered. COWS repeated one hour after first dose. If COWS did not improve by ≥2 points, additional 4mg administered and observed another hour, then discharged home with additional 4mg (max 12 mg on induction day).<br><br>Two 4mg doses provided to take as needed for withdrawal symptoms.	Not reported	Phase 2 mean 20.3mg (SD=7.9, range 8-32mg/day) in a single daily dose (all participants, not just chronic pain)	Tapered

© 2021 Powell VD et al. *JAMA Network Open.*

Webster et al, 2016.	Inpatient	Demonstrate feasibility of switching to buccal buprenorphine without a taper. All patients were physically opioid dependent by naloxone challenge but did not have a history of substance abuse.	Morphine or oxycodone; dose range 80-220 OME/day	8-12 hours	Buccal	Those using 80-160 OME received 300µg buccal buprenorphine q12 hours; those using 161-220 OME received 450µg q12 hours	Rescue medication was available; details not described	600 – 900 µg / day (in two divided doses)	Only provided for duration of study

[1]For studies with multiple interventions or comparator arms, data is only provided for individuals who had chronic pain and were transitioned to buprenorphine from LTOT.

© 2021 Powell VD et al. *JAMA Network Open.*
